# Reweighting UK Biobank corrects for pervasive selection bias due to volunteering

**DOI:** 10.1093/ije/dyae054

**Published:** 2024-05-07

**Authors:** Sjoerd van Alten, Benjamin W Domingue, Jessica Faul, Titus Galama, Andries T Marees

**Affiliations:** School of Business and Economics, Vrije Universiteit Amsterdam, Amsterdam, Netherlands; Tinbergen Institute, Amsterdam, Netherlands; Graduate School of Education, Stanford University, Stanford, CA, USA; Survey Research Center, Institute for Social Research, University of Michigan, Ann Arbor, MI, USA; School of Business and Economics, Vrije Universiteit Amsterdam, Amsterdam, Netherlands; Tinbergen Institute, Amsterdam, Netherlands; Center for Economic and Social Research and Department of Economics, University of Southern California, Los Angeles, CA, USA; School of Business and Economics, Vrije Universiteit Amsterdam, Amsterdam, Netherlands

**Keywords:** Selection bias, volunteer bias, ascertainment bias, collider bias, participation bias, bias correction, UK Biobank, inverse probability weighting

## Abstract

**Background:**

Biobanks typically rely on volunteer-based sampling. This results in large samples (power) at the cost of representativeness (bias). The problem of volunteer bias is debated. Here, we (i) show that volunteering biases associations in UK Biobank (UKB) and (ii) estimate inverse probability (IP) weights that correct for volunteer bias in UKB.

**Methods:**

Drawing on UK Census data, we constructed a subsample representative of UKB’s target population, which consists of all individuals invited to participate. Based on demographic variables shared between the UK Census and UKB, we estimated IP weights (IPWs) for each UKB participant. We compared 21 weighted and unweighted bivariate associations between these demographic variables to assess volunteer bias.

**Results:**

Volunteer bias in all associations, as naively estimated in UKB, was substantial—in some cases so severe that unweighted estimates had the opposite sign of the association in the target population. For example, older individuals in UKB reported being in better health, in contrast to evidence from the UK Census. Using IPWs in weighted regressions reduced 87% of volunteer bias on average. Volunteer-based sampling reduced the effective sample size of UKB substantially, to 32% of its original size.

**Conclusions:**

Estimates from large-scale biobanks may be misleading due to volunteer bias. We recommend IP weighting to correct for such bias. To aid in the construction of the next generation of biobanks, we provide suggestions on how to best ensure representativeness in a volunteer-based design. For UKB, IPWs have been made available.

Key Messages

- We investigated the extent to which volunteer-based sampling of UK Biobank (UKB) biases associations of interest and developed inverse probability (IP) weights to correct for such bias.
- Volunteer bias in all associations, as naively estimated in UKB, was substantial, yet IP weighting reduced volunteer bias substantially, by ∼87% on average. Our constructed weights are made available to UKB users.
- We advise next-generation biobanks to mitigate problems of volunteer bias by collecting variables in common with a data set representative of the target population (e.g. a census), allowing bias estimation and IP weight calculation.

## Introduction

Large-scale biobanks (*N* > 100 000) have become a key resource for medical, epidemiological, genetic and social scientific research.^1–4^ UK Biobank (UKB), used in >3200 peer-reviewed publications,^5^ is a well-known example. Such large samples are vital for the identification of small effects with sufficient power and make, for example, genome-wide association studies feasible.^6^ However, a focus on size has led to a reliance on volunteering at the expense of representativeness.^3^^,^^7–11^ Consequently, such data sets exhibit ‘healthy volunteer bias’ as respondents tend to be healthier and of higher socio-economic status than the population from which they were sampled.^9–11^

It is debated whether such bias challenges subsequent scientific investigations. One view is that representativeness need not be a necessary goal to uncover causal relationships (e.g. the effect of an exposure *X* on an outcome *Y*).^12^^,^^13^ Careful study design ensures that the exposure of interest is unrelated to any other participant characteristics that might influence the outcome, including sample selection. However, exposures of interest are rarely unrelated to other characteristics in observational data.

Understanding an outcome–exposure relationship then typically starts with estimating an association between two variables, possibly controlling for confounding factors. Some claim that generalizable associations can be estimated even in non-representative data.^8^^,^^14–16^ However, in a volunteer-based sample, various types of selection biases may arise, which cause the association of interest to differ from the true association present in the underlying target population.^17^^,^^18^ Under type 1 selection bias, study participation in itself can serve as a ‘collider’ on the path from the exposure to the outcome,^19^ threatening the internal validity of the study.^7^^,^^17–21^ Type 2 selection bias occurs if the effect of the exposure on the outcome is heterogeneous within the target population and selection is based on a variable that modifies the effect of the exposure on the outcome.^17^^,^^22^ Under type 2 selection bias, internal validity may hold but external validity is at risk. Both types of bias could potentially be of concern but type 1 selection bias is potentially the most harmful, as internal validity is necessary for external validity.

**Figure 1.**
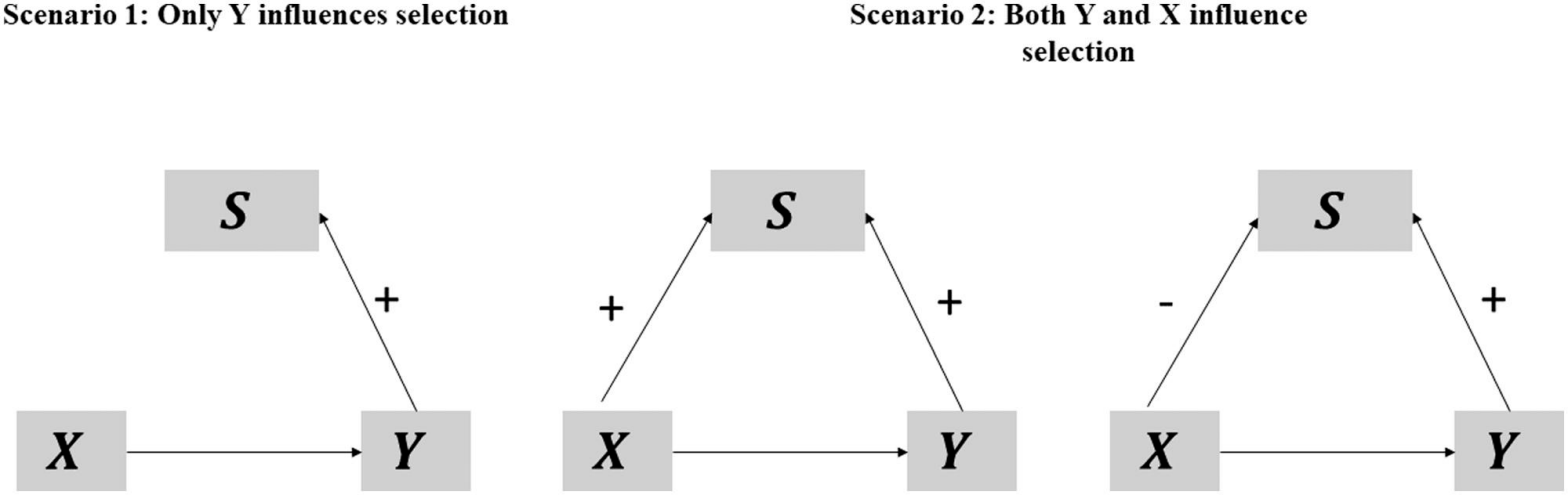
Direct acyclic graphs of selection bias under various scenarios. Here, X refers to the exposure of interest, Y to the outcome variable and S to participation of an individual in the sample in which the relationship between X and Y is estimated. Under all these scenarios, S serves as a collider on the path from X to Y, biasing the estimated relationship between X and Y within the sample. In Supplementary Note S1 (available as Supplementary data at *IJE* online), we use simulations to illustrate the direction of the bias in the estimated exposure–outcome relationship in more detail

In Figure 1, we use direct acyclic graphs to represent possible scenarios under which type 1 selection bias in a volunteer-based biobank can occur. Troublingly, the direction of bias is not known, as it depends on which variables influence selection into the data set and how these variables, in turn, relate to the exposure and outcome (see Supplementary Note S1, available as Supplementary data at *IJE* online).^18^ Understanding how volunteering biases estimates is vital to understanding the costs and benefits of large (but non-representative) vis-à-vis smaller (but more representative) data sets. In addition, methods to correct for such bias are needed.

In this study, we examined the degree of volunteer bias in association statistics estimated in UKB. We used inverse probability (IP) weights, constructed using external, representative data from the UK Census, to correct for volunteering, and evaluated the costs and benefits of volunteer-based sampling.

## Methods

### Data

We followed Rudolph *et al.*^23^ by defining the target population as all individuals who received an invitation to participate in UKB (the ‘UKB-eligible population’). We therefore refer to three data sets: UKB, the UKB-eligible Census and the Weighted UKB.

UKB: Between 2006 and 2010, UKB sent invites to 9.2 million UK citizens aged 40–69 years living in proximity to one of 22 assessment centres.^24^ Only 5.5% participated. UKB respondents are older, more likely to be female and to reside in less socio-economically deprived areas compared with UKB's sampling population.^10^^,^^16^ We dropped a small set of 11 237 UKB respondents (2.2% of the total) for reasons of data quality (Supplementary Note S2, available as Supplementary data at *IJE* online). Supplementary Figure S1 (available as Supplementary data at *IJE* online) illustrates how many UKB respondents we lost at each step. Our final UKB data set includes 491 268 UKB respondents. We study selection into this main sample.

UKB-eligible Census: The census year closest to the UKB assessment period is 2011. The 2011 Census Microdata Individual Safeguarded Sample (Local Authority) for England and Wales^25^ and Scotland^26^ is a random 5% subsample of the 2011 UK Census (N≈3.1 million). It is highly representative due to its high response rate (95%).^25^

**Figure 2.**
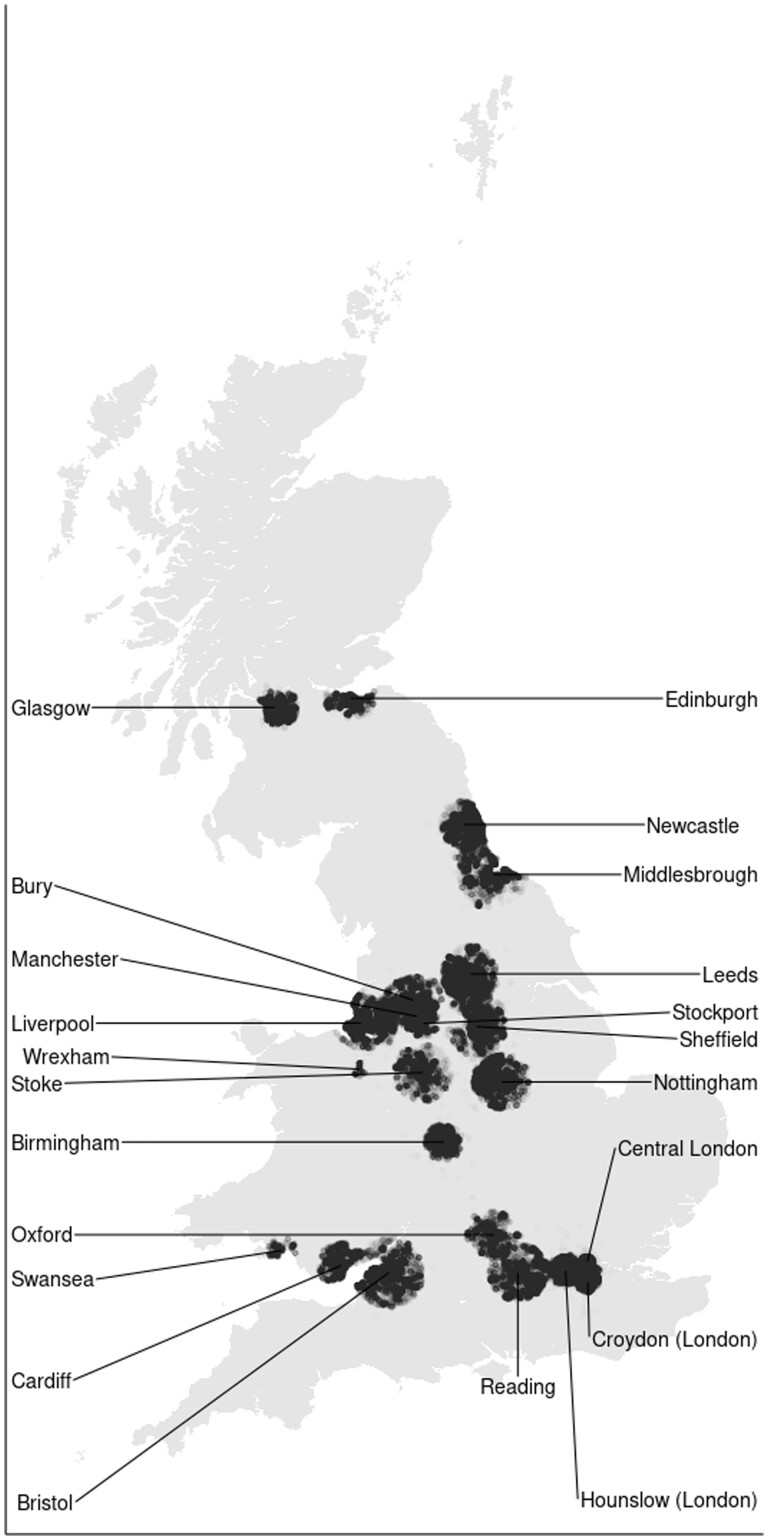
UK Biobank (UKB) respondents’ location of residence at assessment day. Each black dot corresponds to the place of residence of a UKB respondent. Only respondents who lived near any of the UKB assessment centres (annotated), which were predominantly located in urban areas, received an invitation to participate in UKB

Our goal was to make UKB representative of the ‘UKB-eligible population’. The UKB-eligible population differs from the overall UK population in two important ways, as the UKB only sampled individuals who (i) were aged between 40 and 69 years and (ii) lived close to any of 22 assessment centres. These assessment centres were mostly located in urban areas. As Figure 2 shows, this led to an uneven sampling of regions, leaving out large swaths of Great Britain. We therefore restricted the UK Census microdata according to respondents' birth cohort and region of residence, using information on the sampling radii around the 22 assessment centres from which UKB respondents were sampled (Supplementary Note S3, Supplementary Figure S2, available as Supplementary data at *IJE* online). The final sample size of this ‘UKB-eligible Census’ is 687 491.

Weighted UKB: The Weighted UKB is the UKB after application of IP weights (IPWs) to correct for volunteer bias.

### Statistical analysis

To obtain IPWs for UKB respondents, we estimated a probit model that predicts the UKB participation decision on concatenated data from UKB (UKB = 1) and the UKB-eligible Census (UKB = 0). We used predictors based on year of birth, sex, ethnicity, educational attainment, employment status, region of residence, tenure of dwelling, number of cars in the household, self-reported health and one-person-household status. These variables were selected based on two inclusion criteria. First, they had to be assessed for all UKB baseline respondents and UK Census respondents. Second, they had to be assessed using the same (or similar) wording in their respective questionnaires. We harmonized all responses into categories that are comparable in both data sets (Supplementary Note S4, Supplementary Table S1, available as Supplementary data at *IJE* online).

We used exact matching to impute missing variables (Supplementary Note S5, available as Supplementary data at *IJE* online). We entered all selected variables non-parametrically in the model by creating a dummy variable for each level that the variable takes and included all possible two-way interactions between these dummy variables. As a result, we used 4820 predictors. We performed variable selection by using LASSO estimation (see detail in Supplementary Note S6, available as Supplementary data at *IJE* online). The resulting predicted participation probabilities were then used to construct IPWs for all UKB respondents that were inversely proportional to their estimated probability of participation (Supplementary Note S7, Supplementary Figure S3, available as Supplementary data at *IJE* online).

To assess whether volunteer bias affects UKB, we compare means, standard deviations and bivariate linear regression coefficients in UKB and the UKB-eligible Census. We tested the null hypothesis that these coefficients are the same using a Z-statistic: $\frac{{\hat{\beta }}_{\mathrm{UKCensus}}\;-\;{\hat{\beta }}_{\mathrm{UKB}}}{\sqrt{{\hat{se}}_{\mathrm{UKCensus}}^{2}\;-\;{\hat{se}}_{\mathrm{UKB}}^{2}}}$.

Next, we used our IPWs to correct means, standard deviations and regression coefficients in UKB for volunteer bias. Supplementary Table S2 (available as Supplementary data at *IJE* online) lists the formulae used to construct the weighted statistics. To assess whether our IPWs also change associations between variables of interest not available in the Census, we also estimated associations of lifestyle risk factors with all-cause mortality in UKB, using unweighted and IP-weighted Cox proportional hazard models (see Supplementary Note S8, available as Supplementary data at *IJE* online for details). These associations are directly comparable to weighted associations of a previous study of volunteer bias in UKB, which estimated weights based on the Health Survey of England (HSE) rather than the UK Census.^27^ Last, we used two methods to derive an effective sample size for the Weighted UKB (see Supplementary Note S9, available as Supplementary data at *IJE* online), providing an estimate of the size of a population-representative sample with the same power.

## Results

### Differences between UKB and the UK Census are consistent with healthy volunteer bias

A comparison of the UKB-eligible Census and UKB reveals substantial non-random selection of UKB participants from the UKB-eligible population (Table 1). Compared with the UKB-eligible Census, individuals who participated in UKB were older, healthier, more highly educated, of higher socio-economic status and more likely to be White. For all variables included in the table, means differ between UKB and the UKB-eligible Census ($P<10^{-8}$), with some large and quantitatively important differences. For example, individuals in the UKB-eligible Census were over twice as likely to report being in poor health compared with UKB participants (9.3 vs 4.4%), despite the fact that UKB participants are ∼3.5 years older on average.

**Table 1.** Summary statistics for the UK Biobank-eligible Census, UK Biobank and the Weighted UK Biobank^a^

Variable	UKB-eligible Census	UKB	Weighted UKB
**Discrete/continuous variables**			
Age (years) in 2011			
Mean	55.899	59.455	56.181
SD	9.039	8.261	8.901
*n*	687 491	491 268	491 240
Self-reported health			
Mean	2.625	2.701	2.638
SD	0.648	0.546	0.633
*n*	687 489	488 956	488 941
Years of education			
Mean	12.608	13.585	12.892
SD	5.071	4.987	5.042
*n*	687 489	480 251	480 233
No. of cars			
Mean	1.364	1.547	1.392
SD	0.966	0.870	0.962
*n*	683 138	487 832	487 820
**Bivariate variables**			
Female			
Mean	0.508	0.546	0.512
SD	0.500	0.498	0.500
*n*	687 491	491 268	491 240
University or equivalent			
Mean	0.278	0.336	0.290
SD	0.448	0.472	0.454
*n*	687 489	480 251	480 233
Reports ‘poor health’			
Mean	0.093	0.044	0.085
SD	0.290	0.206	0.279
*n*	687 489	488 956	488 941
Has paid work			
Mean	0.609	0.579	0.614
SD	0.488	0.494	0.487
*n*	687 491	486 711	486 693
Retired			
Mean	0.249	0.341	0.250
SD	0.432	0.474	0.433
*n*	687 491	486 711	486 693
Incapacitated			
Mean	0.069	0.033	0.065
SD	0.254	0.179	0.247
*n*	687 491	486 711	486 693
Unemployed			
Mean	0.033	0.016	0.032
SD	0.179	0.127	0.176
*n*	687 491	486 711	486 693
House owner			
Mean	0.736	0.899	0.753
SD	0.441	0.302	0.431
*n*	683 138	484 157	484 147
White ethnicity			
Mean	0.888	0.946	0.893
SD	0.315	0.226	0.309
*n*	687 491	491 268	491 240
One-person household			
Mean	0.181	0.185	0.186
SD	0.385	0.388	0.389
*n*	683 138	487 922	487 908

a For all variables, mean values differ between the UK Biobank-eligible Census and UK Biobank (UKB), all with *P *<* *10^−8^, as obtained by using a Z-test. After applying inverse probability weighting, the means and standard deviations (SDs) of these variables in the Weighted UKB are closer to those of the UKB-eligible Census. *n* refers to the sample size. For the Weighted UKB, *n* refers to the number of respondents with weights available. Small differences in sample sizes between UKB and the Weighted UKB may occur when weights are missing, as 60 respondents could not have all relevant variables imputed using exact matching (see Supplementary Note S5, available as Supplementary data at *IJE* online).

Further, for all four discrete and continuous variables in Table 1, we observe more narrow distributions (smaller standard deviations) in UKB compared with the UKB-eligible Census, consistently with non-random (over)sampling of those more likely to volunteer (see Supplementary Notes S1 and S10, available as Supplementary data at *IJE* online, for additional explanation).

After IP weighting (rows labelled ‘Weighted UKB’), distributions in UKB became much more comparable to those in the UKB-eligible Census, as reflected by more similar means and standard deviations. Hence, IP weighting was successful in correcting the distributions in UKB for volunteer bias. Supplementary Table S1 (available as Supplementary data at *IJE* online) shows the full frequency distributions of these variables in UKB, the UKB-eligible Census and the Weighted UKB.

### Volunteer bias affects associations estimated in UKB

**Figure 3.**
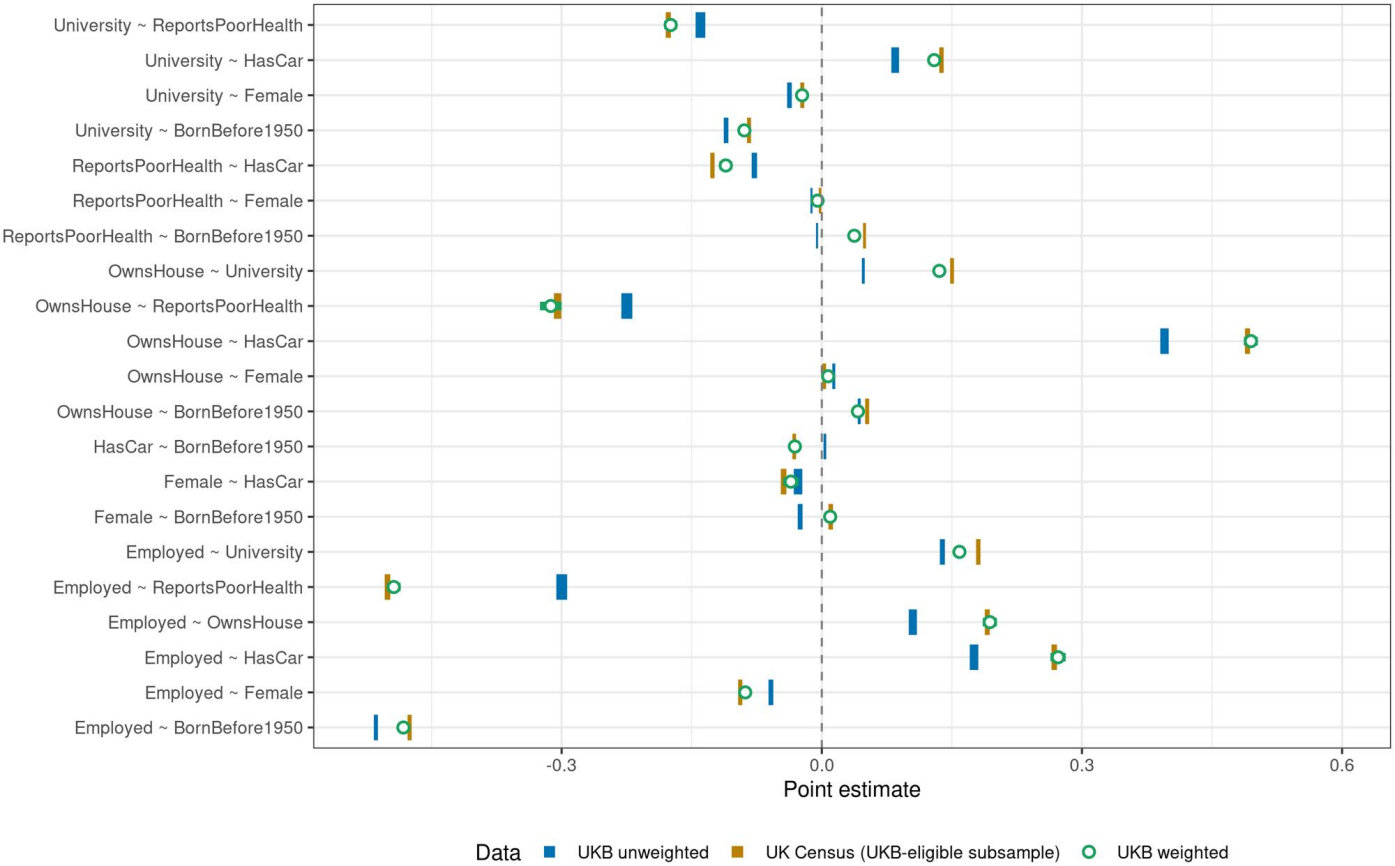
Estimated associations based on bivariate linear probability models in UK Biobank (UKB; solid dark bars), the UKB-eligible Census (solid light bars) and the Weighted UKB (open circles). Bar widths indicate 95% CIs (heteroskedasticity-robust standard errors). All dark and light bars differ from one another ($P\;<\;10^{-8}).$ Inverse probability weighting leads to substantially improved associations: the open circles are in all cases substantially closer to the light bars than the dark bars are to the light bars

We next tested to what extent the associations estimated in UKB are affected by volunteer bias. Figure 3 plots the coefficients of bivariate linear probability models estimated in the UKB-eligible Census (light bars) and UKB (dark bars). The width of these bars indicates the 95% CI. We tested the null hypothesis that each coefficient was the same in the UKB-eligible Census and UKB, and found $P<10^{-8}$ in all cases. Sizeable differences between the associations in both data sets suggests that volunteering biases these association statistics in UKB substantially. For example, the association between being employed and reporting poor health amongst UKB respondents (95% CI = –0.306; –0.294) is substantially weaker than in the UKB-eligible population (95% CI = –0.504; –0.498). Estimating associations in UKB can thus result in sizeable distortions of the actual associations in the underlying target population.

**Figure 4.**
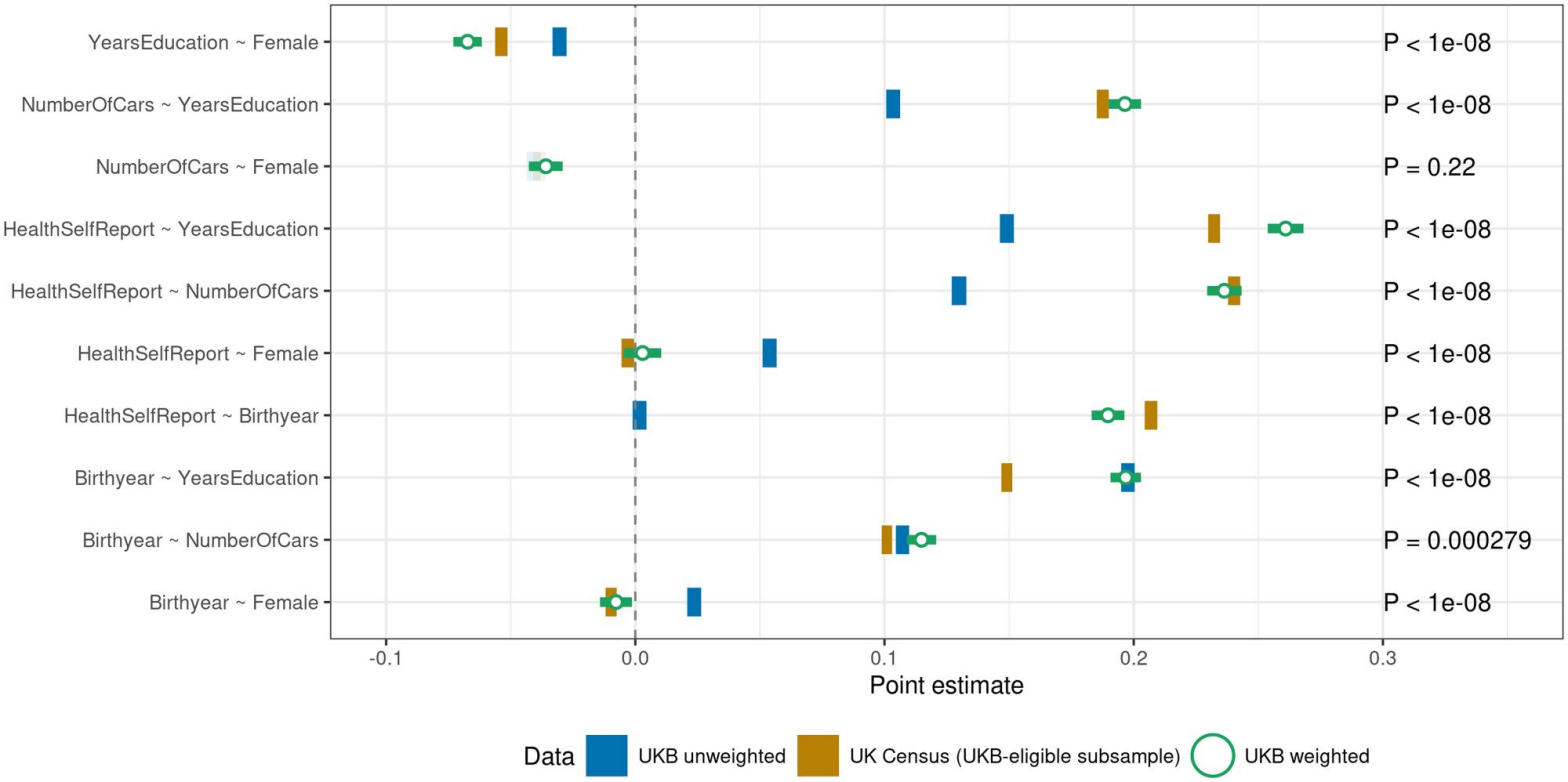
Estimated associations based on linear models in UK Biobank (UKB; solid dark bars), the UKB-eligible Census (solid light bars) and the Weighted UKB (open green circles). All variables are standardized with mean 0 and variance 1. All solid dark and light bars differ from one another (*P*-values shown). Bar widths indicate 95% CIs (heteroskedasticity-robust standard errors). Inverse probability weighting leads to substantially improved associations: the open circles are substantially closer to the light bars than the dark bars are to the yellow bars

Whereas most ‘UKB unweighted’ estimates in Figure 3 are at least in the correct direction, volunteer bias can also lead to false positives or an incorrect sign. For example, UKB individuals born before 1950 were ‘less’ likely (95% CI = –0.007; –0.004) to report being in poor health than younger individuals. Although this association is small, it is contrary to the vast evidence that age is associated with poorer health, and illustrates how volunteer bias can result in misleading associations. Indeed, in the UKB-eligible Census, we observe the expected positive association (95% CI=0.0476; 0.0509). Further, women in UKB were less likely to have been born before 1950 than men (95% CI = –0.0278; –0.0221), whereas the reverse holds in the UKB-eligible Census (95% CI = 0.008; 0.0129), consistently with men living shorter lives than women. We obtained similar results for bivariate linear models between several discrete and/or continuous variables (Figure 4).

### IP weighting is consistent with reducing volunteer bias (variables shared with the UK Census)

IP-weighted regressions correcting for volunteer bias (see open circles in Figures 3 and 4, including 95% CIs) show less biased associations in the Weighted UKB. Weighted estimates are substantially closer to the estimates in the UKB-eligible Census (light bars) compared with the unweighted UKB associations (dark bars). The average bias reduction in estimated associations is 87% over all bivariate linear probability models shown in Figure 3 and 78% over all linear models between discrete and/or continuous variables shown in Figure 4.

Note that including variables that influence UKB participation as linear controls in the regression, rather than weighting, is not a valid way to mitigate volunteer bias, as these variables are colliders when type 1 selection bias is present.^28^ An IP weighting strategy should be used instead. We illustrate this in Supplementary Note S11 (available as Supplementary data at *IJE* online). We also tested whether our IPWs are robust to removing variables from the participation prediction model. One by one, we removed both variables from each of the associations in Figure 3 and re-estimated the model underlying the IPWs (Supplementary Note S12, Supplementary Figure S4, available as Supplementary data at *IJE* online). We conclude that ∼69% of volunteer bias remains controlled for in these associations compared with 87% for the full model.

### IP weighting is consistent with reducing volunteer bias (variables not shared with the UK Census)

Another test of the performance of our IPWs was to evaluate the effect of IP weighting on variables in UKB that were not measured in the Census, and to see whether these corrections were consistent with volunteer bias. We estimated the means of such variables with and without applying IP weighting (Supplementary Table S3, available as Supplementary data at *IJE* online). Findings are consistent with healthy volunteer bias. After weighting, UKB volunteers are younger, heavier, in worse (mental) health, more likely to smoke and of lower socio-economic status. For example, weighting UKB increases the Townsend deprivation index from –1.317 to –0.414, consistently with oversampling of high socio-economic-status individuals (a higher Townsend index indicates lower socio-economic status, with 0 being the UK-wide average). Weighting increases the prevalence of substance use and various health conditions (i.e. reported chest pain, disability), providing additional evidence regarding the oversampling of healthier individuals.

Last, we repeated analyses of weighted and unweighted associations between all-cause mortality and various lifestyle risk factors, allowing comparisons with a previous study of volunteer bias in UKB, which estimated weights based on the HSE rather than the UK Census^27^ (see Supplementary Note S8, Supplementary Figures S5, available as Supplementary data at *IJE* online). Our results are consistent with those of Stamatakis *et al*.,^27^ who find small changes in the associations before and after weighting. However, for physical activity, we find that our weights substantially alter the association with all-cause mortality: no physical activity (compared with >7.5 h of physical activity per week) has Hazard Ratio (HR) = 1.34 before weighting (*P *= 0.008) and HR = 2.03 after weighting (*P *= 0.000 004 8).

### Volunteer bias reduces the effective sample size of UKB to 32% of its original size

We used two methods to arrive at an effective sample size for UKB (Supplementary Note S9, available as Supplementary data at *IJE* online). The first uses the distribution of IPWs and obtains an effective sample size of 200 810 (40.8% of the size of UKB). The second is regression-specific. It results in effective sample sizes for each of the estimated association statistics in Figure 4 that range between 118 370 and 202 999, with an average of 156 698 across all models. Hence, after weighting, the information obtained from the full UKB sample of 491 268 is equivalent to that obtained from a hypothetical representative sample taken from the same target population of between 118 370 and 202 999 individuals (24–41% of the size of UKB).

## Discussion

We uncovered substantial non-random selection of UKB participants by comparing UKB with its target population. Volunteer bias is present in all 21 associations we tested. In some cases, volunteer bias leads to false positive associations or associations that are of the incorrect sign. By constructing IPWs to correct for volunteer bias, we were able to correct for 87% of volunteer bias on average for associations tested between binary variables and 78% between discrete or continuous variables. After IP weighting, variable distributions in UKB become more similar to those in the target population (UKB-eligible Census), providing further evidence of reduced volunteer bias. IPWs also correct associations with variables that were available in UKB but not in the Census in ways that are consistent with volunteer bias. Researchers can use these IPWs in UKB analyses to assess the robustness of estimates to volunteer bias when representative data are not readily available for such comparisons.

Earlier studies of volunteer bias in UKB associations exclusively focused on mortality as an outcome.^16^^,^^27^ These studies used the HSE and the Scottish Health Survey (SHS). For example, Batty *et al*.^16^ compared risk factors for mortality between UKB and the HSE/SHS, and concluded that volunteer bias was of little importance. Stamatakis *et al*.^27^ estimated IPWs using HSE data to correct such risk factors and found some evidence for volunteer bias. We investigated a more comprehensive set of associations between socio-economic and health-related variables, and find that volunteer bias matters substantially. Our study distinguishes itself in at least five ways. First, we compared UKB to the UK Census, which, with a response rate of 95%, is highly representative of the UKB population compared with those of the HSE/SHS, which have a lower mean response rate (68%)^27^ and may therefore not be sufficiently representative due to potential participation bias. Second, the use of rich UK Census microdata allowed us to include many more variables and their interactions (for a total of 4820 predictors), improving precision of the weights compared with previous work. Third, we used fine location information to restrict the UK Census data to UKB’s target population, which resides around 22 highly urbanized areas. By contrast, the HSE/SHS do not contain detailed geographic information. This is of key methodological importance as sufficient overlap between UKB and the target population is key to the validity of the estimation of IPWs.^29^ Fourth, the large sample size of the UK Census aids more precise IPW estimation (687 491 respondents in our final sample compared with 6666 in the HSE used by Stamatakis *et al*.^27^). Last, our weights are estimated using predictors of selection bias that were missing in previous analyses—most importantly, the region of residence, which is one of the strongest predictors of selection into UKB (see Supplementary Figure S6, available as Supplementary data at *IJE* online). As a result, due to our precisely estimated weights, we find that weighting substantially alters association statistics in UKB, unlike previous efforts.

We acknowledge limitations. Our proposed method of IP-weighted regression reduces volunteer bias but increases standard errors. However, this does not necessarily imply a decrease in power, as volunteer bias may take the form of attenuation bias, resulting in larger effect sizes after weighting (see Supplementary Note S1, available as Supplementary data at *IJE* online). Only a limited number of variables—those that UKB and the UK Census have in common—are included in the weights. These are largely related to socio-demographics and health. There may exist unobserved variables that also explain UKB participation, e.g. personality characteristics. Nonetheless, our weights reduce a substantial part (87%) of volunteer bias in UKB-estimated associations. Even when leaving variables out of IPW estimation, we could correct for 69% of volunteer bias on average. Finally, some UKB respondents may also be present in our UK Census subsample and may thus be considered both a respondent and a non-respondent when we predict UKB participation. This results in downward bias of the probability participation estimates, but not in our IPWs because the relative ordering of the weights amongst UKB respondents remains the same as when we would have been able to take UKB respondents out of the UK Census subsample (see Supplementary Note S13, available as Supplementary data at *IJE* online).

Our weights have been returned as a data field to UKB for use in research. We encourage researchers to use these weights in their own analyses of UKB data. A complete mitigation of volunteer bias is not guaranteed. Nonetheless, IPWs are able to greatly reduce volunteer bias compared with unweighted research designs under a wide variety of selection scenarios.^17^^,^^18^ The actual amount of volunteer bias reduced by the weights will depend on the research question and association of interest.

Our findings have implications for biobank study design. Volunteer-based designs may lead to substantial reductions in effective sample size. Biobanks face a choice of whether to follow a volunteer-based sampling scheme that can be adjusted by providing well-estimated sampling weights or to devote considerable resources to obtain a smaller sample that is close to representative. Further efforts are now being made to set up biobanks that oversample members from groups under-represented in research,^30^ hence deviating from representativeness. Such strategies can be advisable, as long as representativeness ‘within’ the oversampled groups is ensured ‘and’ weights are provided that are inversely proportional to the probability of including respondents from each group.

When volunteer-based designs are used, we would suggest that the construction of weights be a prospective design goal and that data be collected such that weights can be readily constructed (e.g. by harmonizing with variables available in representative data, such as a census or population registry). If representative designs are of interest, this may require increasing participation rates through methods such as telephone-based invitations rather than postal-based invitations^31^ or providing (monetary) incentives for study participation.^32^ Another possible avenue might combine volunteer-based sampling with case prioritization to ensure that the types of individuals that are unlikely to respond are prioritized.^33^

## Ethics approval

The UK Biobank study is approved by the National Health Service’s Health Research Authority (ref. 21/NW/0157; UK Biobank Application Number 55154).

## Supplementary Material

dyae054_Supplementary_Data

## Data Availability

UK Biobank data are accessible upon request and approval by the UK Biobank committee (https://www.ukbiobank.ac.uk/). UK Census safeguarded microdata are available from UK Data Service (https://ukdataservice.ac.uk/), upon request and approval. All code used for generating the results is available at https://github.com/sjoerdvanalten/UKBWeightsFinal. The IPWs developed here have been returned to UKB and are available as a data field to UKB-approved researchers.
