# Supplementary material for: Reweighting UK Biobank corrects for pervasive selection bias due to volunteering
Source: Int J Epidemiol. 2024 May 7;53(3):dyae054. doi: 10.1093/ije/dyae054 (PMC11076923; doi:10.1093/ije/dyae054)
Supplement: dyae054_Supplementary_Data [file dyae054_supplementary_data.pdf]

Supplementary Material to: Reweighting the UK Biobank corrects for pervasive selection bias due to volunteering

Contents

**S1**	**Consequences of non-random sampling for associations in non-representative data**	**2**
**S2**	**UKB: Geographic sampling and restrictions to the main sample**	**3**
**S3**	**Restricting and adjusting UK Census data to the UKB-eligible subsample**	**5**
**S4**	**Harmonisation of variable categories across the UKB and UK Census**	**7**
**S5**	**Missing data in the UK Census and UKB, and imputation procedure**	**14**
**S6**	**LASSO estimation of UKB participation probabilities**	**15**
**S7**	**Construction of Inverse Probability Weights**	**17**
**S8**	**Weighted and unweighted estimates between all-cause mortality and life style factors**	**18**
**S9**	**UKB effective sample size**	**19**
**S10**	**Effects of sample selection bias on the standard deviation of bivariate variables**	**21**
**S11**	**Addressing selection bias by adding control variables in the regression**	**22**
**S12**	**Robustness of IP weighted regressions to missing variables**	**23**
**S13**	**The effect of UKB observations present in Census data on weights estimation**	**24**
**S14**	**Supplementary Tables & Figures**	**27**

S1 Consequences of non-random sampling for associations in non-representative data

In this supplementary note, we model how some types of selection biases, illustrated in figure 1, can arise, and assess the direction of these biases. Non-random selection into the data set at hand can arise for a large variety of reasons, and bias the analysis at hand in various ways. The goal of this note is not to provide a complete overview of all types of selection biases, but rather to provide an illustration of how various types of selection biases can bias the analysis in different types of directions. For a more complete overview, we refer the reader to Breen & Ermisch and Lu et al.[17,18]

To illustrate the consequences of non-random selection into a non-representative cohort study such as the UKB, consider the simple case where the effect of an exposure X on an outcome Y is assessed using bivariate linear regression. Figure S7 shows simulations of an exposure $X \sim \mathcal{N}(0, 1)$ and an outcome $Y = X + \epsilon$, $\epsilon \sim \mathcal{N}(0, 1)$, and three scenarios (1, 2a and 2b). In all 3 scenarios, X and Y are positively related in the population (the orange and blue dots combined) with slope 1. This is reflected by the orange regression lines in each of the three scatter plots.

In scenario 1, individuals with higher values of Y, here modelled by a threshold $Y > Y^*$, select into the sample (S; the blue points) and there is no selection based on X. As a result, the regression line estimated within the selected sample S (the blue line) is attenuated towards the null. This attenuation bias occurs irrespective of the sign between X and Y.

In scenario 2a, individuals with higher values of Y and higher values of X, here modelled by a threshold $0.5Y + 0.5X > Z^*$, select into the sample S. As a result, the regression line estimated within the selected sample S is biased downwards. Given the parameters of our simulation, the downward bias is so severe that the regression line is of the incorrect sign.

In scenario 2b, individuals with higher values of Y, but lower values of X, select into the sample S (here modelled by a threshold $Y - 2X > Z^*$). Now, the bias is upwards, and the effect of X on Y is overestimated. Further, when X and Y both correlate with the probability of selection into S, volunteer bias can be introduced even when X and Y are not related to one another in the underlying

sampling population, introducing false positive associations within the sample. Note further that, in all these scenarios, the standard deviations of X and Y estimated within the selected sample S are smaller, compared to the standard deviations in the full population, as a consequence of selection (this can be seen from the distributions of the blue dots in the simulations, which are more narrow).

S2 UKB: Geographic sampling and restrictions to the main sample

We made various restrictions to the full UKB sample for purposes of data quality, resulting in a small loss of 11,237 respondents in total. Supplementary Figure S1 illustrates how many UKB respondents we lost at each step.

First, we dropped those with missing values of sex, region of residence, ethnicity, or year of birth. We also dropped those who died before Census day (March 27th 2011), loosing 2,998 respondents. As such, our definition of the UKB's target population is conditional on survival up until this date. We next removed individuals in the UKB who were sampled by the UKB, but nonetheless did not meet the criteria to be eligible for the UKB-eligible population (those eligible to receive an invite), based on their year of birth or region of residence, as follows.

First, we dropped all UKB participants who were not aged between 40 and 69 at the start of their assessment centre's sampling period. That is, for assessment centres that started sampling in 2007, we kept all individuals born between 1937 and 1967, for assessment centres that started sampling in 2008, we kept all individuals born between 1938 and 1968, etc. However, we kept individuals born in 1969 assessed at the Bristol centre (which started assessment in 2008), and kept individuals born in 1970 assessed at the Birmingham centre (which started assessment in 2009), as these centres both sampled a significant number of individuals born in these years. This resulted in dropping 98 UKB respondents.

The UKB sampled respondents residing close enough around one of the 22 assessment centres. Figure S2 shows the place of residency of all UKB participants on the day they visited the assessment

centre. Some have claimed that UKB assessment centres sent out invites to all individuals in the targeted age range that were living within a radius of 40 km of the assessment centre.[3] Therefore, we dropped 87 UKB participants who lived further than 40 km away from any assessment centre. However, a closer inspection of the data revealed that, for virtually all assessment centres, the sampling radius is considerably smaller, and the size of the sampling area varies per assessment centre. For example, Figure S2 shows that the assessment centre in Edinburgh only sampled respondents in relative proximity (max 22.4 km), whereas the assessment centre in Middlesbrough sent out invitations to a wider area (max 39.9 km).

We obtained the sampling radius of each assessment centre from the UKB data as follows: for each assessment centre, we assumed that the UKB participant who lived furthest away was also the furthest living person to receive an invite for that centre, and we defined the assessment centre's sampling radius accordingly.[1] However, this method is sensitive to outliers. For 14 assessment centres, we obtained sampling radii that were unrealistically large, as the difference between the furthest participant and the participant in the 99.9th percentile of the distance-to-assessment centre distribution was more than 2 km. For these centres, we defined the sampling radius as the 99.7th percentile of distances to the assessment centre, plus 2 km. The resulting sampling radii are visualised as the circles in Figure S2. A small number of UKB respondents (108) fell outside the sampling radii of any assessment centre, and were excluded from our data set.

Last, to ensure that our UKB participation probabilities were robustly estimated, we dropped UKB participants residing in Census grouped local authority (GLA) districts with fewer than 70 UKB participants. This resulted in a small loss of another 263 UKB respondents.

[1]We reassigned individuals who lived within 40 km of an assessment centre, but visited an assessment centre further away, to their nearest assessment centre.

S3 Restricting and adjusting UK Census data to the UKB-eligible subsample

We restricted the Census data to observations that would have recived an invitation to participate in the UKB during its data collection period from 2006 to 2010 (the "UKB-eligible population"), using information on their year of birth and region of residence.

First, we only kept individuals aged 40 to 74 at the time of the Census, as these could have been aged between 40 and 69 between 2006 and 2010. We used the age of the individual on the day of the Census (5-year bins) to infer the year of birth bin of each individual. We restricted this age range further using adjustment factors, created as discussed below.

Second, we determined the regions of residence the UKB included in its sampling population, as follows. Location of residence in the UK Census data is reported at a higher level of aggregation than in the UKB, namely by *grouped local authority* (GLA) *region* (grouped council authority; GCA, for Scotland, named GLA here for convenience). These regions consist of a single local authority when the population in these regions was larger than 120,000, and of aggregated groups of neighbouring local authorities otherwise. There are 285 distinct GLA regions covering all of Great Britain. We then restricted the UK Census data to only include those Census individuals that resided in a GLA region that falls *at least partially* within the sampling radius of one of the 22 assessment centres in England, Wales, or Scotland (see Figure S2 and supplementary note S2). We then used adjustment factors that account for the share of the area that falls within the UKB sampling regions, i.e. around the assessment centres, as well as the share of the population that is in the UKB-eligible age range. The way we created these is discussed below.

Weighting UK Census data using adjustment factors: Restricting the UK Census based on age and region as described above introduces some individuals in our sample that may not have received a UKB invite during the period 2006 to 2010, because they were not in the relevant age range (40 to 69) at the time that their nearest assessment centre started sampling, or because they

lived too far from a UKB assessment centre. We therefore calculated adjustment factors to ensure that our UK Census sample is as representative as possible of the UKB-eligible population in 2006 and 2010, conditional on survival up until March 2011. To start, we assigned to each individual in our UK Census data set an initial adjustment factor of 20, to upweight the 5% random subsample to the full UK population. We next adjusted these adjustment factors in the following two ways.

First, individuals who resided in GLA regions that did not fully fall within the UKB sampling radius of any assessment centre had their adjustment factor multiplied by the proportion of the population living within this GLA region that is within the sampling radius (see supplementary note S2 for additional details on the estimation of the assessment-centre specific sampling radii). These GLA-specific sampling population proportions are calculated using 2011 UK Census population counts reported for the much less aggregated *lower layer super output areas* (LSOAs) for England and Wales. For Scotland, even less aggregated *output areas* (OAs) are used because LSOAs are unavailable. For England and Wales, there are 34,753 distinct LSOAs and for Scotland, there are 46,351 OAs. Figure S2 shows the GLA regions that are included in the final sample, and illustrates how adjustment factors are assigned to each Census respondent living in these regions (the adjustment factors range from adjustment factors of 0 [white] to 20 [black] and anything in between [grey]).

Second, the year of birth distribution of the UKB is assessment-centre specific, as some assessment centres started sampling sooner than others (supplementary note S2). For example, the assessment centre in Manchester sampled all its respondents in 2007, and hence only sampled respondents born between 1937 and 1967, as these were aged 40 to 69 at the time. By contrast, the centre in Swansea sampled all its respondents in 2010, and hence only sampled respondents born between 1940 and 1970. We faced the extra challenge that the UK Census only reports year of birth bins of 5 years. As a result, the year of birth distribution of our UKB-eligible UK Census sample and the UKB do not necessarily overlap, i.e., not everyone in the year of birth bins 1936-1940 or 1966-1970 (at the edges of the UKB-eligible distribution) was UKB-eligible. For Census individuals in these year of birth bins, we assigned them to an assessment centre based on

the GLA in which they live. We multiplied their adjustment factor by the proportion of year of birth values (out of a maximum of five) in this year of birth bin that were sampled by the assessment centre. For example, Census respondents residing in or around Manchester that were born between 1936-1940 had their sampling weight multiplied by 0.8, since Manchester only sampled those with year of birth 1937, 1938, 1939, and 1940, but not 1936. When a GLA region overlapped with the sampling radius of multiple assessment centres, we took the largest possible adjustment factor.

The final UKB-eligible census subsample consists of 687,491 observations. Our adjustment factors for this sample range between 0.22 and 20 with a mean of 15.8 and a median of 18.6. This implies a UKB-eligible population of 10,836,059 individuals (summing all sampling weights), slightly larger than the true number of invitations that were sent out by the UKB (9,238,452). Throughout this paper, all statistics reported on the *UKB-eligible population* (Table 1, Figure 3, Figure 4, Table S1, Table S4, Figure S4, Figure S9, Figure S10) are estimated on this subsample and weighted using the adjustment factors.

In Table S4, we compare the UKB-eligible subsample of the UK Census to the full UK population of this age range (i.e. all Census data). Compared to the full population, the UKB-eligible population is more ethnically diverse, younger, of lower socioeconomic status (as measured by an overall deprivation indicator), more urbanised (as measured by various proxies for urbanicity), and in worse health. These differences are relatively small compared to those induced by participation in the UKB.

S4 Harmonisation of variable categories across the UKB and UK Census

To estimate our IP weights, estimate our summary statistics on the UKB and the UKB-eligible Census, and estimate regression models in both these data sets, we selected variables that were assessed in a similar fashion in the UKB and the UK Census, allowing for comparisons. When needed, we altered the categorisation of several variables in the UKB and UK Census to ensure

that the variables were comparable across both data sets.

S4.1 Year of Birth

- **UK Census:** Year of birth was derived from 5-year age bins that described the age of the individual at the day of the UK Census (40-44; 45-49; 50-54; 55-59; 60-64; 65-69; and 70-74). These were recoded into the following year of birth values: (1966-1970; 1961-1965; 1956-1960; 1951-1955; 1946-1950; 1941-1945; 1936-1940).[2]

- **UKB:** We recoded year of birth into the 5-year of birth bins as mentioned above.

S4.2 Sex

- **UK Census:** Respondents were asked "What is your sex?", and could answer male or female.

- **UKB:** Sex as recorded by the NHS registry, but possibly updated by the participant.

S4.3 Region of residence

- **UK Census:** Region of residence was inferred from the respondent's address. The respondent's address was prefilled by the Census data collectors, and corrected by the respondent when necessary. Region of residence is reported as 265 distinct grouped local authorities (GLA; England & Wales), and 20 grouped council areas (GCA; Scotland). GLAs or GCAs consist of local authorities and council areas, or groups of adjacent local authorities and council areas to ensure that each GLA/GCA has at least 120,000 inhabitants.

- **UKB:** Coordinates of home location at assessment, inferred from NHS registry data on the postcode level, rounded to the nearest kilometer. These coordinates were aggregated into the

[2]The 2011 UK Census was conducted at 27th of March. We classify each respondent in their 5 year of birth bin assuming that they had *not yet* had their birthday in 2011. This approach inevitably results in some small classification error (e.g., someone who turned 65 on February 1st of 2011 has year of birth 1946, is classified in age bin 65-69 by the UK Census, and is next, erroneously, classified in year-of-birth-bin 1941-1945 by us).

same GLAs and GCAs mentioned above, using .shp files that describe the borders of these GLAs and GCAs, obtained from the office for national statistics.

S4.4 Ethnicity

- **UK Census:** Respondents were asked "What is your ethnic group?", and could respond "White", "Mixed", "Asian/Asian Birtish", "Black/African/Caribbean/Black British", or "Other"
- **UKB:** Wording of the question was the same as in the UK Census. The available categories were also the same, except that "Chinese" was recognised as a separate ethnic group. We merged this group with "Asian/Asian British". Additionally, respondents could answer "None of the above" or "Prefer not to answer". We coded these responses as missing.

S4.5 Economic status

- **UK Census:** UK Census respondents were asked what their main economic activity was last week through various questions. Answers were coded by the Census bureau into the following categories:

1 Economically Active (excluding Full-time students), In Employment, Employee, Part-time

2 Economically Active (excluding Full-time students), In Employment, Employee, Full-time

3 Economically Active (excluding Full-time students), In Employment, Self employed with employees, Part-time

4 Economically Active (excluding Full-time students), In Employment, Self employed with employees, Full-time

5 Economically Active (excluding Full-time students), In Employment, Self employed without employees, Part-time

6 Economically Active (excluding Full-time students), In Employment, Self employed without employees, Full-time

7 Economically Active (excluding Full-time students), Seeking work and ready to start within 2 weeks, and Waiting to start a job already obtained and available to start within 2 weeks

8 Economically Active Full-time students, In employment

9 Economically Active Full-time students, unemployed, seeking work and ready to start within 2 weeks, and waiting to start a job already obtained and available to start within 2 weeks

10 Economically Inactive, Retired

11 Economically Inactive, Student

12 Economically Inactive, Looking after home/family

13 Economically Inactive, Permanently sick/disabled

14 Economically Inactive, Other

We recoded these levels into a sparser number of categories, namely "Employed" (1-6, 8), "Retired" (10), "Stay-at-home" (12), "Incapacitated" (13), "Unemployed" (7), and "Student" (9, 11)

- **UKB:** At baseline, UKB respondents were asked in the touchscreen questionnaire: "Which of the following describes your current situation?". Participants could respond to this question as follows:

1 In paid employment or self-employed

2 Retired

3 Looking after home and/or family

4 Unable to work because of sickness or disability

5 Unemployed

6 Doing unpaid or voluntary work

7 Full or part-time student

-7 None of the above

-3 Prefer not to answer

We classified those doing unpaid or voluntary work as “Stay-at-home”, and coded those answering “None of the above” or “Prefer not to answer” as missing.

S4.6 Tenure of household

- **UK Census:** Respondents answered the question: “Does your household own or rent this accommodation?” They could answer that they own it outright, own it with a mortgage or loan, part own and part rent (shared ownership), rent, or live rent-free.

- **UKB:** Respondents answered the question: “Do you own or rent the accommodation that you live in?” If respondents tapped the “Help” button they were shown the following:

  “Please select: - Own outright if you or someone in your household owns the accommodation that you live in. - Own with mortgage if you or someone in your household has a mortgage on the accommodation that you live in.”

  Answer categories were the same as in the UK Census, except that respondents could also answer “None of the above”, or “Prefer not to answer”. We coded these as missing.

S4.7 Number of vehicles in the household

- **UK Census:** Respondents answered the question: “In total, how many cars or vans are owned, or available for use, by members of this household?” (Please include company vehicles if available for private use). They could answer none, 1, 2, 3, or 4 or more.

- **UKB:** The wording of this question was identical to the wording in the UK Census, as were the answering categories. In addition, we coded those answering “None of the above” or “Prefer not to answer” as missing.

S4.8 Household size

- **UK Census:** The only variable regarding household size available to us in the UK Census data was a dummy that indicated whether the person resided in a one-person household or not.

- **UKB:** Respondents answered the question;"Including yourself, how many people are living together in your household? (Include those who usually live in the house such as students living away from home during term, partners in the armed forces or professions such as pilots". We recoded the answers into a dummy variable that described whether the answer was 1, or some higher number. In addition, we coded those answering "None of the above" or "Prefer not to answer" as missing.

S4.9 Self-reported health

In the UK Census, respondents answered the question "How is your health in general?". In the UKB, respondents answered the question "In general how would you rate your overall health?". Self-reported health in the UKB was assessed through a 4-level Likert scale (Poor, Fair, Good, Excellent), whereas self-reported health in the 2011 UK Census was assessed through a 5-level scale (Very Bad, Bad, Fair, Good, Very Good). To minimise classification error, we harmonise these values in both data sets to a three-level scale (Bad, Fair, Good/Excellent), by combining the categories "Very Bad" and "Bad" in the UK Census, and lumping Good/Excellent/Very good into a single category. In addition, respondents in the UKB could answer "Do not know" or "Prefer not to answer", these answers were coded as missing.

S4.10 Education

In the UK Census, respondents were asked "Which qualifications do you have?" and were instructed to tick every box that applied. These answers were recoded by the Census bureau into the highest level of education obtained for each respondent. In the UKB, respondents were asked the question

“Which of the following qualifications do you have? (You can select more than one)”. However, the potential answers to each question differed between the UK Census and the UKB. We harmonised the level of education across both data sets as follows:

- **UK Census:** The UK Census assigned International Standard Classification of Education (ISCED) levels to the variable “highest degree obtained”. We assigned years of education based on these levels: 7 for no degree (assuming primary school), 10 for a level 1 or level 2 degree, 13 for a level 3 degree, and 20 for a level 4+ degree. This assignment follows previous work in the UKB.[4] Two other categories in the UK Census were not assigned any ISCED level in the UK Census data. These were “apprenticeship” and “Other: Vocational/Work-related qualifications, etc.”. We assigned 12 years to the “apprenticeship” (reflecting the fact that it requires continuing one’s education after a GCSE-degree, but does not require an A/AS-level degree).[5] For the “vocational” category, we again followed previous work,[4] and assigned 15 years of education.

- **UKB:** For the UKB, we similarly assigned years of education to degree categories that clearly fall within the categories recognised by the UK Census. These categories are: “No degree" (ISCED1), “College or university” (ISCED4+), “A levels/AS levels or equivalent” (ISCED3), “O levels/GCSEs or equivalent” (ISCED2) and “CSEs or equivalent” (ISCED2). However, for those who reported having a “National Vocational Qualification (NVQ) or Higher National Diploma (HND) or Higher National Certicificate (HNC) or equivalent” or “Other professional qualifications, e.g. nursing, teaching”, assigning years of education was not as straightforward. For those holding an NVQ, we do not know which level of NVQ certificate they hold (In the UK Census, an NVQ of level 1 is considered an ISCED1 degree, whereas an NVQ of level 4 or higher is considered ISCED4+). Accordingly, substantial heterogeneity in the variable “age at which left full-time education” can be seen for the group of respondents holding a degree in this category, with a substantial group of respondents having left full-time education before the age of 16 (Figure S8a). To solve this issue, for those holding an NVQ (or HND or HNC) we assigned years of education by taking the age at

which the respondent reported to have left full-time education, minus 5, and capped the value at 19 years of education.[6] For those holding a professional degree, we similarly observed substantial heterogeneity in the age at which these respondents left full-time education (Figure S8b). Hence, for this group, we estimated years of education in similar fashion, but capped the variable at 15 years. When UKB respondents reported multiple degrees, we took the maximum of years of education associated with each degree.

These continuous years of education measures make educational attainment comparable across the UKB and the UK Census. For estimating the selection model, we discretized the variable. Those less than 8.5 years of education get level 1 (no degree), those between 8.5 and 11 get level 2 (O-levels or equivalent), those between 11 and 17.5 level 3 (A-levels, vocational, or equivalent), and those above 17.5 get level 4 (college/university, or equivalent).

S5 Missing data in the UK Census and UKB, and imputation procedure

Both the UK Census and UKB had missing data on the variables we use to predict UKB participation. Table S5 provides an overview for each variable we use. In the UKB, respondents explicitly had the option to not share information on all variables measured through self-reporting. They could either tick the options "prefer not to answer" or "do not know". We coded such values as missing. As a result, 4.9% of our included UKB respondents had missing data on at least one predictor included in the selection model. In the UK Census, data on the variables we use is typically not missing, but for some variables regarding the household in which the individuals live (i.e., tenure of dwelling, number of cars owned, and household size), no information is available for 0.63% of the observations, as these were people living in communal establishments.

Our model uses a large number of regressors to predict UKB selection status. 24,380 UKB and 4,355 UK Census observations had at least one regressor missing. These missing values are imputed using an exact matching procedure. We conduct exact matching by converting the data

to a frame that holds the following variables: whether it was a UK Census or UKB data point, region, year of birth, sex, education, self-reported health, employment status, ethnicity and sex. In step one, we fill in missing values by sampling from observations with the exact same values on all these variables. For observations for which an exact match could not be found, we attempted to match again using the same variables, but dropping region and ethnicity from the data frame. For 60 observations, this procedure did not yield an exact match, such that IP weights could not be estimated.

S6 LASSO estimation of UKB participation probabilities

We model the likelihood of participating in the UKB for individual i, conditional on having received an invitation, $Pr(UKB = 1|Z_i')$, as

$$Pr(UKB = 1|Z_i') = \Phi(\alpha + Z_i'\delta + \nu_i), \tag{1}$$

with $\Phi(.)$ the standard normal cumulative distribution function (CDF), α a constant, ν_i a random error term, and Z_i' a vector of variables that influences one's individual propensity for participating in the UKB. Variables included in Z_i' are sex, year of birth (5-year cohort), education level, ethnicity, region of residence (Census GLA), tenure of dwelling, employment status, number of cars in the household, a dummy indicating whether the person lives in a single-person household, and self-reported health. These variables are included in a non-parametric manner (i.e., we use dummy variables for each category of the categorical variables under consideration). Furthermore, we include all possible two-way interactions between these dummy variables as predictors. In total, Z_i' contains 4,820 variables.

We estimate equation 1 by weighted probit regression on the training sample of stacked UKB and UKB-eligible UK Census data, where we assign the outcome variable $UKB = 1$ to each UKB observation, and $UKB = 0$ to each UKB-eligible Census observation. Before we estimate our model, we divide our data set in folds that each hold 20% of the data. We then repeat the estimation

procedure 5 times. At each step, one of these folds is not used in training the model, and the probability of selection is estimated in this holdout fold. As such, we make sure that the model used in constructing the weights is not overfitted on the data. To further prevent overfitting, we estimate the model using a LASSO variable selection procedure.[7] The LASSO model maximises the log-likelihood function of the regular probit, subject to the absolute value of the sum of the coefficients being smaller than a certain constant (as determined by a penalisation parameter λ). This additional constraint in the optimisation problem prevents overfitting of the data when many regressors are included, as it ensures that coefficients of variables that are insufficiently predictive of selection are being shrunk to zero. We estimate our LASSO probit model using *glmnet*,[8] which solves the optimisation problem

$$(\hat{\alpha}, \hat{\delta}) = \arg\min \Bigg\{ \sum_{i=1}^{N} w_i \Bigg(\text{UKB}_i \ln\big(\Phi(\alpha + \sum_j \delta_j z_{ij})\big) + (1 - \text{UKB}_i) \ln\big(1 - \Phi(\alpha + \sum_j \delta_j z_{ij})\big) \Bigg) + \lambda \sum_j |\delta_j| \Bigg\},$$

where w_i is the weight (1 for UKB observations, and the adjustment factor constructed as described in section S3 for UK Census observations), and λ is the penalisation parameter.

The penalisation parameter is chosen through cross-validation using k-folding with 5 folds. The k-folding procedure ensures that λ is chosen as to yield an optimally predictive model on a holdout sample not used in the estimation of the LASSO model. For the model used on the first holdout sample, this results in a penalisation parameter of 0.000026. 568 out of the 4,820 variables we include had their coefficients shrunk to zero. This low penalisation parameter implies that the solutions to our model lie very close to those of a similarly specified regular probit model in which the same variables (including the two-way interactions) are included. These statistics were very similar for the models that held out the other 4 holdout samples of our data.

Our LASSO probit model adequately discriminates between UKB and UK Census observations, with an area under the curve (AUC) of 0.772[9] ($IMV = 0.006$)[10] when holding out the first holdout

sample.[3] For comparison, our AUC is similar to the AUC achieved when predicting mortality in the Health and Retirement Study to correct for mortality selection bias.[11] Figure S6 shows a variable importance plot in which we assess the extent to which the model's performance in the holdout sample degrades when permuting each variable in the sample, one at a time. Through each permutation, the respective variable becomes unrelated to the model's outcome, and hence the relative contribution of that variable to the model's performance can be assessed: the larger the reduction in AUC after leaving the variable out, the more important that variable is to the model's performance. The variables region, year of birth, and education drive most of the performance of the LASSO model. All variables that we include are relevant predictors for UKB volunteering. year of birth, region of residence, and education have the most predictive power.

S7 Construction of Inverse Probability Weights

Inverse probability weighting (IPW) is a method to correct for volunteer bias in observational data.[12–14] We construct inverse probability weights as

$$\mathrm{IPW}_i = \frac{\mathrm{Pr}(\widehat{\mathrm{UKB} = 1})}{\mathrm{Pr}(\widehat{\mathrm{UKB} = 1}|Z_i')} \tag{2}$$

.

where $\mathrm{Pr}(\widehat{\mathrm{UKB} = 1})$ is the average probability of being sampled in the UKB as estimated on the full weighted stacked UKB and UKB-eligible Census, and $\mathrm{Pr}(\widehat{\mathrm{UKB} = 1}|Z_i')$ is the probability of UKB participation for UKB participant i as predicted by the LASSO probit model.

A known issue with the estimation of inverse probability weights using a rich set of predictors is that such rich models may yield some values of $\mathrm{Pr}(\widehat{\mathrm{UKB} = 1_i}|Z_i')$ that are very close to zero, with excessively large values of IPW_i as a result. Such excessive weights can result in noisy estimates of weighted regression coefficients and hence dilute power.[14] We deal with this issue by winsorising our distribution of estimated weights, setting any values of IPW_i lower than the 1st percentile equal

[3]For the other 4 holdout samples, these statistics were very similar

to the value at the first percentile, and any values of IPW_i higher than the 99th percentile equal to the value at the 99th percentile. Figure S3 visualises the distribution of these weights.

S8 Weighted and unweighted estimates between all-cause mortality and life style factors

We calculated the association between all-cause mortality and various life style factors as follows. Similar to previous research that addressed the effects of selection bias on all-cause mortality risk estimates[15] we used a Cox proportional hazard model to estimate mortality risk, mutually adjusting for the following four life style factors: alcohol use, smoking, physical activity, and vegetable and fruit intake. Date of death is available in UKB due to linkage with the National Health Service. All four lifestyle risk factors were reported at baseline.

Alcohol use was coded as never, previous drinker, < 5 drinks per week, or ≥ 5 drinks per week. Smoking was coded as never, current, or previous smoker. Physical activity was reported in the amount of hours per week, and was coded as None, < 7.5 hours, and ≥ 7.5 hours. Fruit and vegetable intake was constructed as the sum of servings of the following: cooked vegetables (one serving is one tbsp), salad and raw vegetables (one serving is one tbsp), fresh fruit (one serving is one piece), and dried fruit (one serving is one piece). We additionally controlled for highest degree obtained (four categories), sex, and age at baseline. The presumably healthiest category of each risk factor was used as the reference variable (i.e., never drinker, never smoked, ≥ 7.5 hours of physical activity, and > 10 Fruit and vegetable servings.)

Prior to estimation, we removed those with missing data on any of these lifestyle risk factors or additional control variables, or those with missing IP weights. Note that those with missing weights included those who died before Census day (March 27th 2011, see Methods). We also removed those with a prior history of major cardiovascular events or cancer at baseline, as recorded by ICD10 codes, as keeping those respondents in the analyses could result in bias. Our final sample consisted of N = 322,598 respondents, amongst which 5,766 mortality cases. To understand whether the

associations between each of these life style factors and all-cause mortality are biased by selection into the UKB, we estimated unweighted and weighted versions of the model. The time scale was age.

The resulting association estimates are reported in Supplementary Figure S5. Weighted mortality risk associations tend to differ from their unweighted counterparts. For example, we find that, after weighting, a supposedly protective effect of alcohol use (i.e. drinking ≥ 5 drinks per week) on mortality is no longer different from zero. The other associations suggest that the effect of other risk factors (fruit and vegetable intake, physical activity, and smoking) on all-cause mortality tend to be underestimated because of selection in the UKB, as our weighted hazard ratios tend to be higher than their unweighted counterparts. The difference made by weighting is especially pronounced for the associations between low levels of physical activity and all-cause mortality: we find that a weighted analysis reveals a much larger association with all-cause mortality than an unweighted analysis. For example, no physical activity (compared to ≥ 7.5 hours a week) has a hazard ratio with mortality of 1.34 ($CI_{95} = [1.079; 1.664]$) in an unweighted analysis, and 2.03 ($CI_{95} = [1.497; 2.743]$) in a weighted analysis.

These types of associations have been estimated in previous research using the UKB, using a different set of weights (based on the Health Survey of England).[15] As such, the performance of our IP weights can be directly compared to this previous effort. This previous effort found that weights similarly moved the association between alcohol use and all-cause mortality towards zero. However, this research also found that weighting made little difference for the effect of physical activity, in contrast to the analyses using our weights.

S9 UKB effective sample size

Our IP-weighting procedure is necessary to reduce volunteer bias in estimated means, variances, and associations in the UKB, but it increases confidence intervals, and therefore reduces power. The effective sample size reflects the power of the weighted UKB as equivalent to a hypothetical

random sample of size $\hat{n}$. We use two methods to estimate $\hat{n}$. Both give similar results.

The first method estimates $\hat{n}$ using the distribution of the IP weights. This measure of effective sample size reflects the weighted sample's ability to uncover the sampling population's mean and variance.[16]

$$\hat{n} = \sum_{i=1}^{n} \lambda IPW_i, \; \lambda = \frac{\sum_{i=1}^{n} IPW_i}{\sum_{i=1}^{n} IPW_i^2} \tag{3}$$

Using the IP weights that we estimated, we find that $\hat{n}$ = 200,810. The attractiveness of this measure is that it is not dependent on any particular regression model. However, it is designed to summarise the amount of information that the data reveals for estimation of means and variances only, and not for regression coefficients. Hence, we next use an additional regression-based measure of the equivalent sample size.

A regression-based equation for $\hat{n}$ can be obtained by rewriting the regular OLS formula for the coefficient's standard errors.[17] Because weighted regression results in wider standard errors than OLS, this measure of $\hat{n}$ represents the size of a representative sample that one would need in order to identify the same coefficient with the same amount of precision using OLS. Hence, we measure effective sample size using the following formula:

$$\hat{n} = \frac{var(\epsilon)}{se(\hat{\beta})^2 * var(x)} \tag{4}$$

This measure of $\hat{n}$ is regression-specific (i.e., it depends on which variables are included), as this influences the value of $se(\hat{\beta})$, $var(\epsilon)$, and $var(x)$. Our models with binary outcomes are not well-suited to calibrate an effective sample size, since the hypothetical case of homoskedastic errors (on which this equation for $\hat{n}$ relies) does not hold for linear models with binary outcomes. Thus, we estimate $\hat{n}$ only on models with discrete outcomes of three levels or more, or models with continuous outcomes (i.e., all models in Figure 4). For these models, $\hat{n}$ ranges between 118,370 and 202,999 with an average of 156,698. Hence, volunteer bias in the UKB reduces the effective sample size of the UKB to by 156,698/491,268 = 31.9% of its actual sample size. Thus, a representative

data set of 156,698 contains as much information as the non-representative sample of 491,268 UKB respondents.

Some caveats are in order. First, formula 4 assumes that the error term in the model is homoskedastic, and that any heteroskedasticity in the error term is introduced because of weighting the data, and not because of characteristics of the true underlying data generating process. It could be that some of the models included in Figure 4 exhibit heteroskedastic errors, even in representative data. However, heteroskedasticity does not seem to drive the sharp reduction in effective sample size that we observe here. When applying the formula for $\hat{n}$ on the *unweighted* UKB coefficients, it matters little whether we use robust ($\bar{\hat{n}}$=484,468) or non-robust ($\bar{\hat{n}}$ =487,304) standard errors. In both cases, the formula produces estimates that are close to the true sample size of the unweighted UKB (491,268).

Another caveat is that our estimate for $\hat{n}$ can only be interpreted as the sample size in equally informative representative data if our weights are able to capture *all* of the volunteer bias. This caveat pertains to the first method of estimating $\hat{n}$ as well. As we discuss in the Discussion, our weights may suffer from omitted variables that also influence UKB volunteering. As a result, some volunteer bias may remain even for association estimates that use our IP weights. Because of this potential of missing variables, a representative sample of size $\hat{n}$ is likely desirable to the weighted UKB. In other words, missing variables in the construction of our weights result in an overestimate of $\hat{n}$: the true effective sample size of the UKB is likely lower.

S10 Effects of sample selection bias on the standard deviation of bivariate variables

Note that for bivariate variables, it is not possible to tell whether an increase or a decrease in the standard deviation in the UKB, vis à vis the standard deviation in the UKB-eligible population, is consistent with selective sampling. This is because, for bivariate variables, the standard deviation is $\sqrt{p(1-p)}$, with p the mean of the variable. Hence, the standard deviation is largest for $p = 0.5$.

When selection into the UKB is such that the mean of the variable becomes closer to 0.5 (from above or from below), the standard deviation will be larger in the UKB than in the UKB-eligible population. For example, this is the case in Table 1 for "University or equivalent": in the UKB-eligible population, 27.8% holds such a degree, whereas in the UKB, this is 33.6%. This change in the mean of the variable is consistent with selective sampling in the UKB (where healthy and higher educated citizens are more likely to participate in scientific studies), but nonetheless results in a larger standard deviation of this variable in the UKB.

S11 Addressing selection bias by adding control variables in the regression

Typically, researchers do not explore outcome-exposure relationships through bivariate models, but include control variables to adjust for possible confounding factors. However, introducing control variables that are also correlated with participation into the data set can exacerbate rather than mitigate bias, as these variables are potential colliders.[18] We illustrate this within our models. First, we re-estimate all the models in figure 3, including sex and year of birth as control variables. Year of birth enters linearly. The models in figure 3 that had the variable "born before 1950" or "female" as the dependent or independent variable were now excluded. The new models are shown in figure S9. As can be seen, the estimates in the UKB and UKB-eligible Census still differ substantially from one another, as is also the case when the controls are not included. We estimate the average volunteer bias after introducing these linear controls, and compare it to the average volunteer bias of the same models in figure 3. By introducing these controls, volunteer bias *increases* on average by 20%. By contrast, IP weighting these models with controls still performs well and reduces volunteer bias by 88% on average, which is very comparable to the 87% volunteer bias reduction in figure 3.

As a second test to show that including control variables is no panacea, we re-estimate the model that regresses the probability of reporting poor health on being born before 1950, after including a

much wider range of possible control variables. In figure S10 we report these coefficients without controls (as in figure 3) and after including the following controls: sex, years of education, number of cars owned, a single household indicator, tenure of household (4 dummy variables), employment status (5 dummy variables), ethnicity (4 dummy variables), and region (142 dummy variables). As can be seen from the figure, adding these richer controls does *not* succeed in changing the negative association of age on reporting poor health towards a positive one in the UKB. By contrast, IP weighting does succeed in flipping the sign, and getting the point estimate closer to the one obtained in the UKB-eligible population, both in the model with and without additional control variables.

S12 Robustness of IP weighted regressions to missing variables

The models that we show in Figure 3 are based on variables that we also include in our LASSO model that underlies the estimation of the IP weights. Hence, our approach potentially overstates the extent to which these IP weights can be expected to mitigate volunteer bias across various types of association models: missing variable bias may be introduced when one tries to use these weights to weight models based on variables that were not included in the IP weighting procedure, reducing the potency of the IP weights.

To confirm that our weights are robust to such missing variable bias, we re-estimate the IP weights for each of the models presented in Figure 3. For each association model, we re-estimate our LASSO model, but leaving out *both* the dependent and the independent variable. Using this approach, we create "leave-variables-out" IP weights to weight each linear association model that we consider. As such, we can assess the performance of our IP weighting procedure when relevant model variables are not included in the weighting scheme. We only perform this sensitivity analysis on the first fold of our data (i.e., a 20% subsample), to reduce the computational burden of re-estimating our LASSO model 21 times.

Figure S4 shows the same results as in Figure 2, but now also includes weighted UKB estimates that use the newly created leave-variables-out weights. In general, the point estimates that are

estimated using the leave-variables-out weights (red open circles) are very similar to those that are based on the weights with all variables (green open circles). We confirm that, even when using the leave-variable-out weights, our IP-weighting procedure reduces volunteer bias as averaged over all models included in the figure by 69% (as compared to 87% in the full model).

The finding that weights estimated on different sets of variables result in very similar point estimates, and also result in substantial bias reduction, is encouraging. This means that our IP-weights are robust to missing variable bias, but are also robust to other forms of bias that may arise, for example, when there are subtle differences in assessment of variables between UKB and UK Census.

S13 The effect of UKB observations present in Census data on weights estimation

We assume the Census to be fully representative of the UKB's target population. As a result of this assumption, it is expected that a subset of respondents in the UKB are also present in the 5% subsample of the UK Census that we use in weight estimation. Because our Lasso probit model assigns $UKB = 0$ to *all* observations coming from the Census, this leads to a type of misclassification. In this supplementary note, we will show that, although such misclassification biases the estimates of UKB participation probabilities in our Lasso Probit model towards the null, the bias in the resulting inverse probability weights is negligible.

Let N be the number of individuals in the Census, and n be the number of respondents in the UKB. In our framework, the probability of UKB participation is different for each type of individual i and driven by a set of observables in x_i, such that $prob(UKB_i) = p(x_i) := p_i$, with $E[p_i)] = 0.055$, as the UKB had a 5.5% response rate overall. As a simplification, we assume that X_i is sufficiently sparse such that multiple people can have the same values in X_i. Then, let N_i and n_i be the number of individuals with X_i in the population and the UKB, respectively. Then, the correct probabilities p_i are

$$p_i = \frac{n_i}{N_i} \tag{5}$$

However, in practice our estimated probabilities $\widehat{p(x_i)}$ come from probit estimates with n_i individuals *also* being classified as Census observations with $UKB = 0$, such that

$$\widehat{p(x_i)} = \frac{n_i}{N_i + n_i} \tag{6}$$

such that our estimates of participation probabilities are always biased downwards ($\widehat{p(x_i)} < p_i$). Rewriting both formulas, we obtain

$$\widehat{p(x_i)} = \frac{p_i}{1 + p_i} \tag{7}$$

We note two observations:

1. Although $\widehat{p(x_i)} \neq p_i$, their relative orderings for different values of x_i remains the same, as $\widehat{p(x_i)}$ is monotonically increasing in p_i for $p_i \in [0, 1]$

2. At sufficiently low values of p_i the bias in $\widehat{p(x_i)}$ due to misclassification becomes negligible: estimates of $\widehat{p(x_i)}$ are relatively more biased for those with a high probability of selection into the UKB, than those with a low probability.

Note that in practice, we construct weights of the form $w(x_i) = \frac{K}{\widehat{p(x_i)}}$, K is a constant that does not impact any weighted proportions in the UKB data, and we use as K the average estimated participation rate $K = \frac{n_i}{N_i + n_i} = 0.052$ (such that the weights have mean one). This implies that our weights are of the form $w(x_i) = \frac{K}{\frac{p_i}{1+p_i}} = \frac{K}{p_i} + K$.

Hence, each weight is slightly increased compared to their "true" counterparts. However, the relative ordering of weights in the UKB remains the same, regardless of whether $\widehat{p(x_i)}$ or the true p_i are used in their construction. Further, the minimum and maximum weights amongst our UKB IP weights are 0.1642 and 7.6125, respectively. According to the formula for $w(x_i)$, this implies values of p_i of 0.463 and 0.00688, respectively, such that the "true" weights should have been

$0.055/0.463 \approx 0.119$ and $0.055/0.00688 \approx 7.99$. Hence, the misclassification problem results in weights that are very similar compared to a case where the misclassification problem would not exist, and results in a compressed distribution of our weights, similar to the winsorizing adjustment that we made to make the weights more precise (see supplementary note S7).

S14 Supplementary Tables & Figures

		Census (UKB-eligible)		UKB		UKB Weighted	
Variable	Levels	N	%	N	%	N	%
Sex	Male	5,333,695.06	49.22	222,971	45.39	231,439.87	48.85
	Female	5,502,364.27	50.78	268,297	54.61	242,331.69	51.15
	Total	10,836,059.32	100.00	491,268	100.00	473,771.56	100.00
Birthyear	1936-1940	674,012.26	6.22	33,586	6.84	27,335.05	5.77
	1941-1945	1,289,775.68	11.90	101,081	20.58	60,011.63	12.67
	1946-1950	1,671,907.91	15.43	114,996	23.41	76,488.43	16.14
	1951-1955	1,624,169.90	14.99	82,433	16.78	73,500.49	15.51
	1956-1960	1,874,898.22	17.30	70,732	14.40	82,886.40	17.50
	1961-1965	2,151,457.40	19.85	61,117	12.44	92,685.17	19.56
	1966-1970	1,549,837.96	14.30	27,323	5.56	60,864.39	12.85
	Total	10,836,059.32	100.00	491,268	100.00	473,771.56	100.00
Education	None	2,920,933.24	26.96	83,553	17.01	114,113.60	24.85
	Lower secondary	2,832,405.03	26.14	141,037	28.71	122,798.62	26.74
	A-levels/vocational	2,069,303.74	19.10	94,150	19.16	88,911.07	19.36
	University	3,013,416.20	27.81	161,511	32.88	133,366.72	29.04
	Total	10,836,058.20	100.00	480,251	100.00	459,190.01	100.00
Ethnicity	White	9,625,885.91	88.83	464,757	94.60	423,104.72	89.31
	Mixed	105,552.61	0.97	2,891	0.59	4,823.58	1.02
	Asian/Asian British	656,703.51	6.06	11,273	2.29	25,786.08	5.44
	Black/Black British	356,016.68	3.29	7,879	1.60	15,672.11	3.31
	Other	91,900.62	0.85	4,468	0.91	4,385.07	0.93
	Total	10,836,059.32	100.00	491,268	100.00	473,771.56	100.00
Health (self-reported)	Bad	1,003,746.72	9.26	21,711	4.42	39,886.38	8.49
	Fair	2,054,220.11	18.96	102,875	20.94	90,094.01	19.17
	Good/Very Good	7,778,091.38	71.78	364,370	74.17	339,912.06	72.34
	Total	10,836,058.20	100.00	488,956	100.00	469,892.44	100.00
Employment status	Paid employment	6,602,507.40	60.93	281,812	57.36	287,283.61	61.46
	Retired	2,693,524.53	24.86	165,944	33.78	116,967.98	25.02
	Stay-at-home	385,636.64	3.56	13,607	2.77	15,748.72	3.37
	Incapacitated	751,258.09	6.93	16,066	3.27	30,387.59	6.50
	Unemployed	359,497.74	3.32	7,998	1.63	15,049.81	3.22
	Student	43,634.92	0.40	1,284	0.26	2,027.17	0.43
	Total	10,836,059.32	100.00	486,711	100.00	467,464.87	100.00
No. of vehicles in household	0	1,992,849.34	18.50	42,717	8.70	80,927.49	17.24
	1	4,350,708.42	40.40	204,076	41.54	190,083.04	40.49
	2	3,243,366.45	30.12	185,911	37.84	145,310.75	30.95

		Census (UKB-eligible)		UKB		UKB Weighted	
**Variable**	**Levels**	**N**	**%**	**N**	**%**	**N**	**%**
	3	881,116.19	8.18	41,968	8.54	39,536.74	8.42
	4 or more	301,482.86	2.80	13,160	2.68	13,637.94	2.90
	Total	10,769,523.25	100.00	487,832	100.00	469,495.95	100.00
Tenure of dwelling	Owns house (no mortgage)	3,812,493.95	35.40	254,890	51.88	169,398.20	36.46
	Owns house w/ mortage	4,109,126.28	38.16	180,172	36.67	180,793.92	38.91
	Shared ownership	49,412.74	0.46	1,428	0.29	2,280.21	0.49
	Rent	2,707,334.15	25.14	44,185	8.99	108,020.06	23.25
	Rent-free	91,156.12	0.85	3,482	0.71	4,155.62	0.89
	Total	10,769,523.25	100.00	484,157	100.00	464,648.01	100.00
One-person household	No	8,822,644.06	81.92	397,792	80.97	381,621.94	81.36
	Yes	1,946,879.19	18.08	90,130	18.35	87,414.88	18.64
	Total	10,769,523.25	100.00	487,922	100.00	469,036.83	100.00

Table S1: **Full summary statistics for the UKB-eligible population. the UKB, and the weighted UKB**. The UKB-eligible population is created by restricting and weighting the 5% subsample of the 2011 UK Census. Summary statistics for the weighted UKB were created using the wtd.table() function of the questionr R package.

**Statistic**	**Formula**
Weighted Mean	$\bar{x} = (\sum IPW_i \cdot x_i)/(\sum IPW_i)$
Weighted Standard Deviation	$\bar{sd} = \sqrt{\left[\sum IPW_i \cdot (x_i - \bar{x})^2\right] / [\sum IPW_i]}$
Confidence interval (95%) around weighted mean	$CI = \bar{x} \pm \frac{\bar{sd}}{\sqrt{N}} \times z_{0.95/2}$
Weighted regression coefficient	Weighted least squares, using IPW_i as the weight

Table S2: An overview of formulas used for weighted statistics, using IP weights to ensure robustness against volunteer bias in the UKB

Table S3: Mean of various variables in the UKB before and after IP weighting: These variables are available in UKB data, but not in the UK Census. The weighted mean gives an indication of what the mean of each variable looks like in the UKB's sampling population. 95% confidence intervals around each mean included

**Variable**	**Mean [95% CI]**	**Weighted Mean [95% CI]**	**% Change**
Anthropometric			
Height	**168.713** [168.684;168.741]	**169.123** [169.094; 169.153]	0.2%
BMI	**27.415** [27.4;27.429]	**27.67** [27.654; 27.685]	0.9%
Waist Circumference	**90.319** [90.277;90.361]	**90.916** [90.873; 90.959]	0.7%
Waist Hip Ratio	**0.871** [0.871;0.872]	**0.876** [0.875; 0.876]	0.5%
Hip Circumference	**103.451** [103.423;103.479]	**103.614** [103.584; 103.644]	0.2%
Demographic			
Age at Recruitment	**56.54** [56.518;56.563]	**53.517** [53.493; 53.541]	5.3%
Urban	**0.862** [0.861;0.863]	**0.875** [0.874; 0.876]	1.5%
Died after Census Day	**0.035** [0.034;0.035]	**0.036** [0.035; 0.036]	2.5%
Early lifetime			
Breastfed	**0.723** [0.722;0.725]	**0.682** [0.68; 0.683]	5.8%
Multiple Birth	**0.023** [0.022;0.023]	**0.024** [0.024; 0.025]	6.3%
Birth Weight	**3.319** [3.316;3.321]	**3.311** [3.309; 3.314]	0.2%
Adopted as a child	**0.015** [0.014;0.015]	**0.016** [0.016; 0.017]	12.4%
Maternal smoking	**0.292** [0.291;0.294]	**0.298** [0.297; 0.3]	2%
Food/Beverage consumption			
Tea	**3.411** [3.403;3.419]	**3.406** [3.397; 3.414]	0.1%
Bread Consumed	**0.849** [0.846;0.852]	**0.835** [0.833; 0.838]	1.6%
Cooked Veg. Consumption	**2.723** [2.718;2.729]	**2.685** [2.679; 2.691]	1.4%
Cheese Consumption	**2.523** [2.52;2.526]	**2.451** [2.448; 2.454]	2.8%
Health			
Disability	**0.289** [0.288;0.29]	**0.303** [0.301; 0.304]	4.8%
Asthma	**0.127** [0.125;0.129]	**0.134** [0.132; 0.136]	5.6%
DBP	**82.259** [82.225;82.293]	**82.023** [81.989; 82.058]	0.3%
SBP	**140.21** [140.148;140.273]	**138.281** [138.22; 138.343]	1.4%
Vitamin D	**48.695** [48.632;48.757]	**46.149** [46.086; 46.213]	5.2%
Calcium	**2.38** [2.38;2.38]	**2.378** [2.378; 2.379]	0.1%
Cholesterol	**5.696** [5.693;5.699]	**5.597** [5.594; 5.6]	1.7%
White Blood Cell Count	**6.882** [6.876;6.888]	**7.014** [7.008; 7.02]	1.9%
Red Blood Cell Count	**4.517** [4.516;4.518]	**4.544** [4.543; 4.546]	0.6%
Chest Pain	**0.162** [0.161;0.164]	**0.188** [0.187; 0.189]	15.6%
Hand grip strength (left)	**29.535** [29.503;29.567]	**30.053** [30.02; 30.086]	1.8%
Hand grip strength (right)	**31.672** [31.64;31.703]	**32.172** [32.139; 32.205]	1.6%
Health behavior			
Ever Smoked	**0.602** [0.6;0.603]	**0.608** [0.607; 0.61]	1.1%
Alcohol Freq.	**4.143** [4.139;4.148]	**3.972** [3.967; 3.976]	4.1%

Number of Cigarettes	**18.331** [18.274;18.387]	**18.723** [18.665; 18.782]	2.1%
Ever Addicated	**0.06** [0.059;0.061]	**0.074** [0.073; 0.075]	23.2%
Ever Smoked Cannabis	**0.442** [0.437;0.447]	**0.542** [0.537; 0.547]	22.5%
Max Freq. Cannabis use	**1.656** [1.645;1.666]	**1.733** [1.722; 1.744]	4.7%
Mental Health			
Depression	**0.277** [0.274;0.28]	**0.295** [0.292; 0.298]	6.6%
Other			
Left Handed	**0.093** [0.093;0.094]	**0.095** [0.094; 0.096]	1.8%
Ambidextrous	**0.017** [0.017;0.018]	**0.019** [0.019; 0.019]	10.4%
Happiness	**4.575** [4.571;4.579]	**4.519** [4.515; 4.523]	1.2%
Socioeconomic			
Townsend Cont.	**−1.317** [−1.326;−1.308]	**−0.417** [−0.427; −0.407]	68.3%
No. of people in household	**2.437** [2.433;2.441]	**2.607** [2.603; 2.611]	7%
Age completed full-time educ.	**16.718** [16.71;16.726]	**16.725** [16.716; 16.733]	0%
Time employed current job	**12.933** [12.894;12.973]	**11.911** [11.873; 11.949]	7.9%
Length working week	**35.256** [35.209;35.304]	**36.135** [36.088; 36.181]	2.5%
Heavy manual work	**1.552** [1.548;1.555]	**1.645** [1.641; 1.648]	6%
Household income	**44830.202** [44731.179;44929.225]	**43496.523** [43396.867; 43596.18]	3%

Table S4: Mean of various variables in the full UK Census 5% safeguarded subsample (ages 40-74), and UKB-eligible subsample of this Census. 95% confidence intervals around each mean included.

**Variable**	**Mean in full pop. [95% CI]**	**Mean in UKB-eligible pop. [95% CI]**
Demographic		
Age	**55.098** [55.082;55.115]	**54.77** [54.747;54.794]
Living in a couple	**0.719** [0.718;0.72]	**0.689** [0.688;0.69]
Born outside UK	**0.119** [0.119;0.12]	**0.164** [0.163;0.165]
White	**0.916** [0.916;0.917]	**0.87** [0.869;0.871]
Sex	**0.509** [0.508;0.51]	**0.507** [0.506;0.509]
Household size	**2.61** [2.608;2.613]	**2.649** [2.646;2.653]
Socioeconomic status		
Deprived in education dimension	**0.264** [0.264;0.265]	**0.264** [0.263;0.265]
Deprived in employment dimension	**0.154** [0.153;0.155]	**0.179** [0.178;0.18]
Deprived in health and disability dimension	**0.357** [0.356;0.358]	**0.371** [0.369;0.372]
Deprived in housing dimension	**0.082** [0.082;0.083]	**0.098** [0.098;0.099]
Deprivation indicator (total)	**1.857** [1.856;1.859]	**1.912** [1.91;1.915]
Years of education	**12.652** [12.643;12.66]	**12.643** [12.629;12.656]
Owns house	**0.407** [0.406;0.408]	**0.361** [0.36;0.362]
No. of cars	**1.463** [1.462;1.465]	**1.366** [1.363;1.368]
Health		
Self-reported health	**3.96** [3.958;3.962]	**3.91** [3.908;3.913]
Disability	**0.235** [0.234;0.236]	**0.247** [0.246;0.248]
Number of housecarers in household	**0.335** [0.334;0.336]	**0.347** [0.345;0.348]
No. in household with illness/disability	**0.449** [0.448;0.45]	**0.468** [0.466;0.47]
Employment		
Employed	**0.612** [0.611;0.613]	**0.613** [0.612;0.614]
Retired	**0.222** [0.222;0.223]	**0.216** [0.215;0.217]
Unemployed	**0.053** [0.053;0.053]	**0.04** [0.039;0.04]
Ever worked	**0.81** [0.809;0.811]	**0.866** [0.864;0.867]
Urbanicity		
Persons per room	**0.407** [0.407;0.407]	**0.424** [0.424;0.425]
Goes to work by public transport	**0.113** [0.112;0.114]	**0.167** [0.166;0.168]
Goes to work by car/motorcycle	**0.642** [0.641;0.643]	**0.635** [0.633;0.637]
Lives in flat or appartment	**0.135** [0.134;0.135]	**0.163** [0.162;0.164]
Religion		
Has no religion	**0.2** [0.2;0.201]	**0.182** [0.181;0.183]
Christian	**0.674** [0.673;0.674]	**0.667** [0.666;0.668]
Other religion	**0.126** [0.125;0.127]	**0.151** [0.15;0.152]
Observations	1 277 785	565 994

Variable	UK Census	UKB
Sex	0%	0%
Education	0%	2.24%
Region	0%	0%
Year of Birth	0%	0%
Health (Self-reported)	0%	0.47%
Tenure of dwelling	0.63%	1.45%
Employment status	0%	0.93%
Number of cars	0.63%	0.7%
One-person household	0.63%	0.68%
Ethnicity	0%	0%

Table S5: Prevalence of missing observations in the UKB-eligible Census and UKB (after restricting the sample as shown in Figure S1)

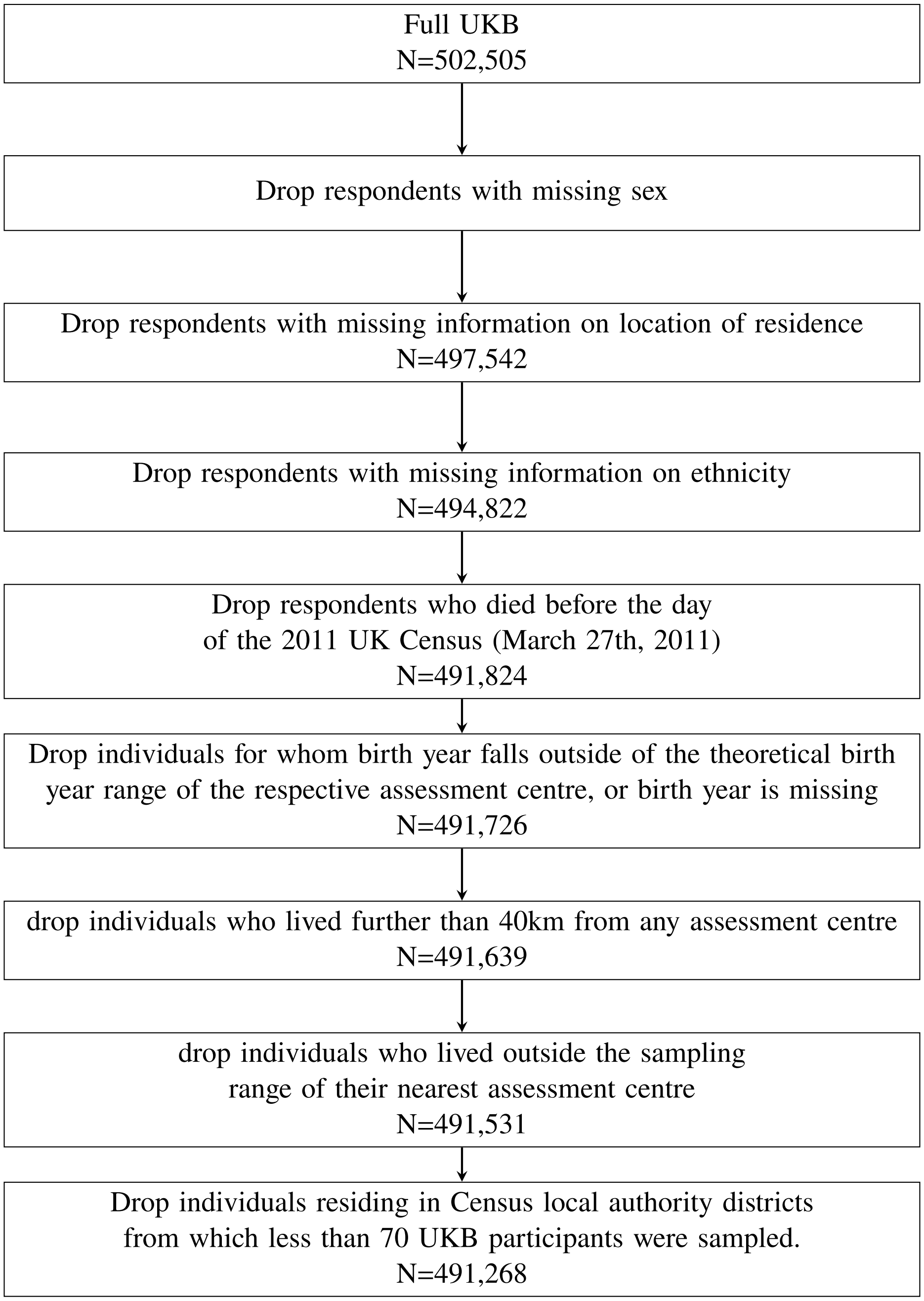

Figure S1: Summary of sample restrictions made to the UKB cohort prior to IPW estimation. N refers to the number of remaining UKB respondents after conducting each sample selection step. Because the number of UKB respondents with missing sex is less than 30, we do not provide a precise value of N because of privacy concerns.

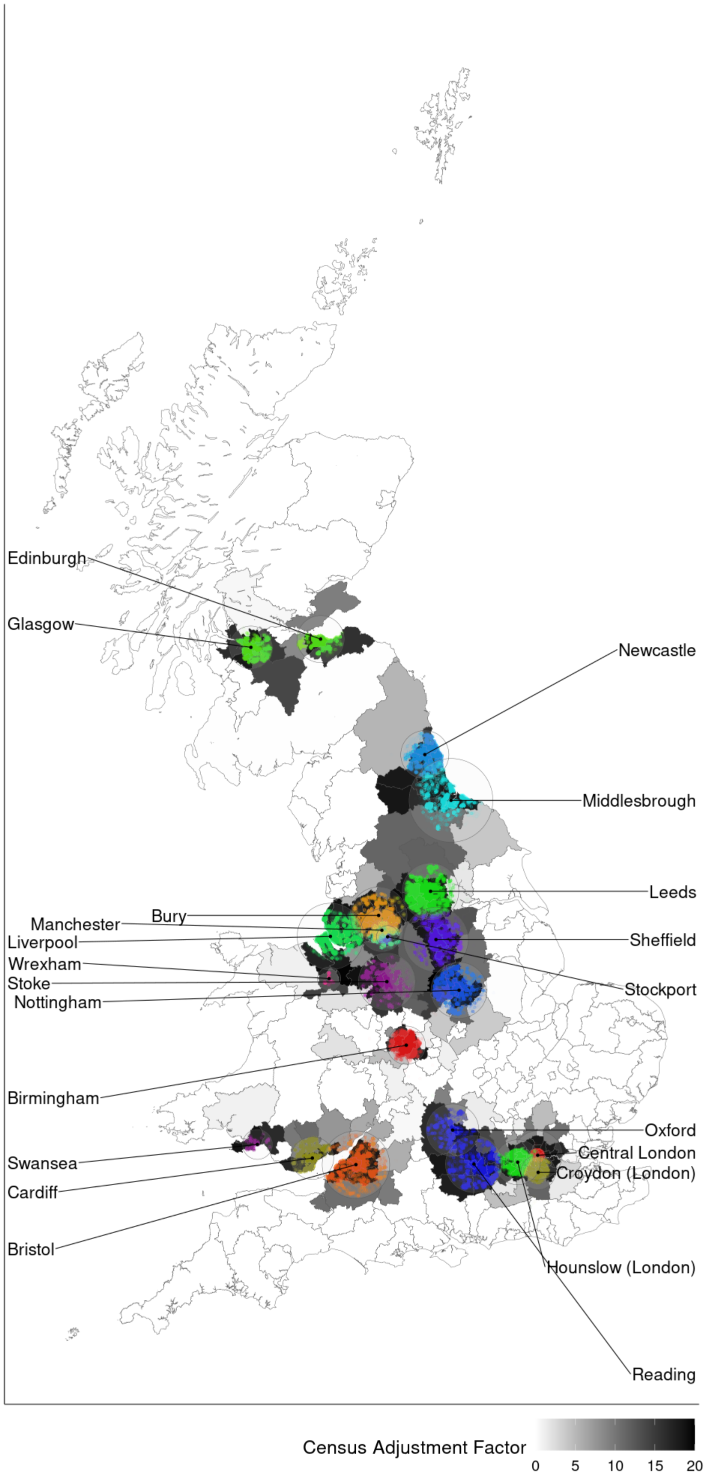

Figure S2: Geographic distribution of UKB participants and the UKB-eligible Census. Each dot on the map represents the geographic location of a UKB participant's residence, coloured by the assessment centre that they visited. Each black dot shows the location of an assessment centre. Each circle visualizes the inferred sampling radius around each assessment centre. Census Grouped Local Authority (GLA) regions that are included in the UKB-eligible Census are coloured in grey. For Census observations from GLA regions that are fully within any assessment centre's sampling region, we assign a sampling weight of 20 (darkest grey shades in the map). For UKB-eligible Census observations from GLA regions that fall only partially within an assessment centre's sampling region, we use adjustment factors of 20 times the share of this region's population that lives within the assessment centre's radius (lighter grey shades in the map).

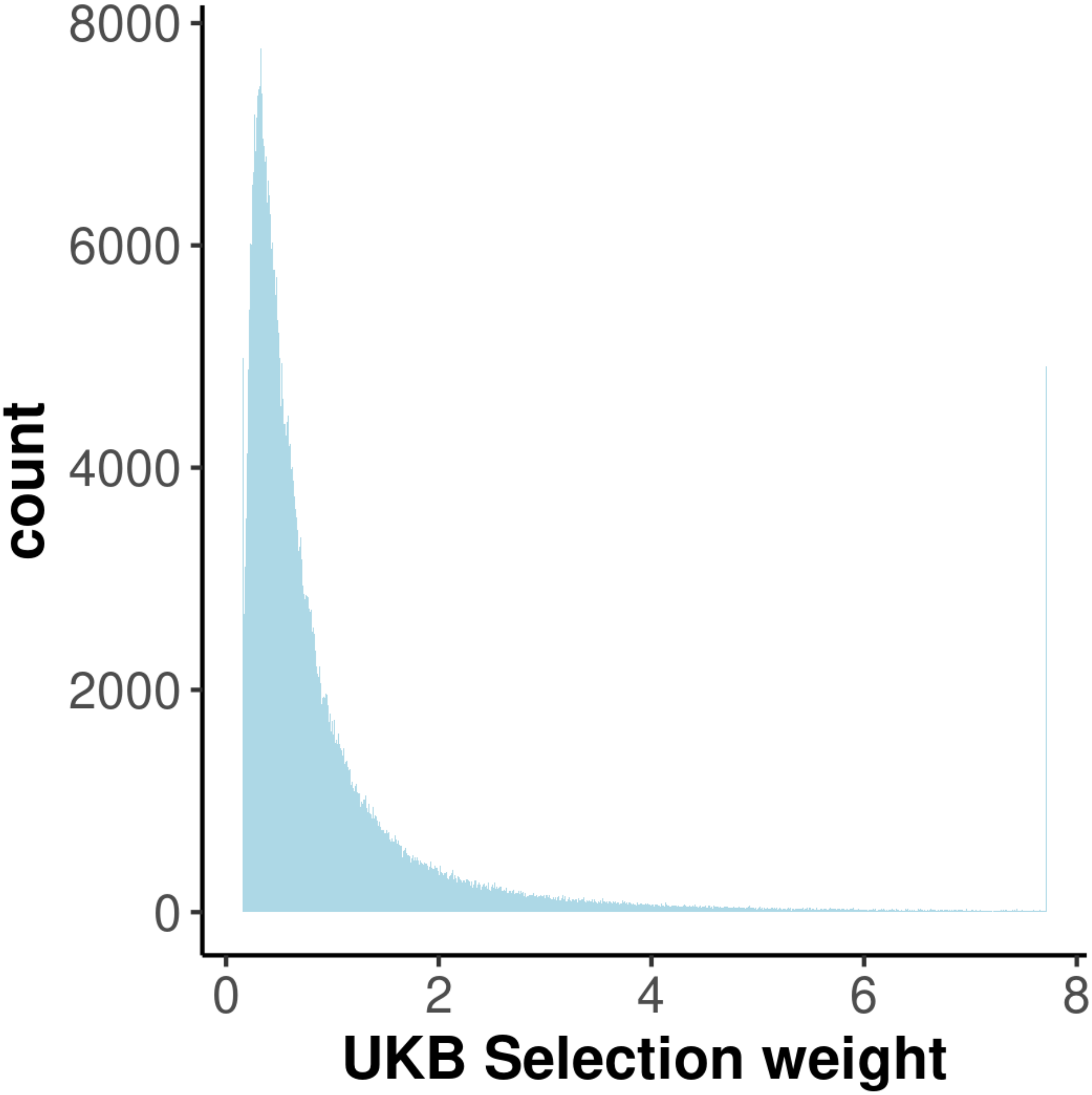

Figure S3: Histogram of the distribution of UKB IP weights after winsorising (setting values below the 1st percentile equal to the value at the 1st percentile, and values above the 99th percentile equal to the 99th percentile.)

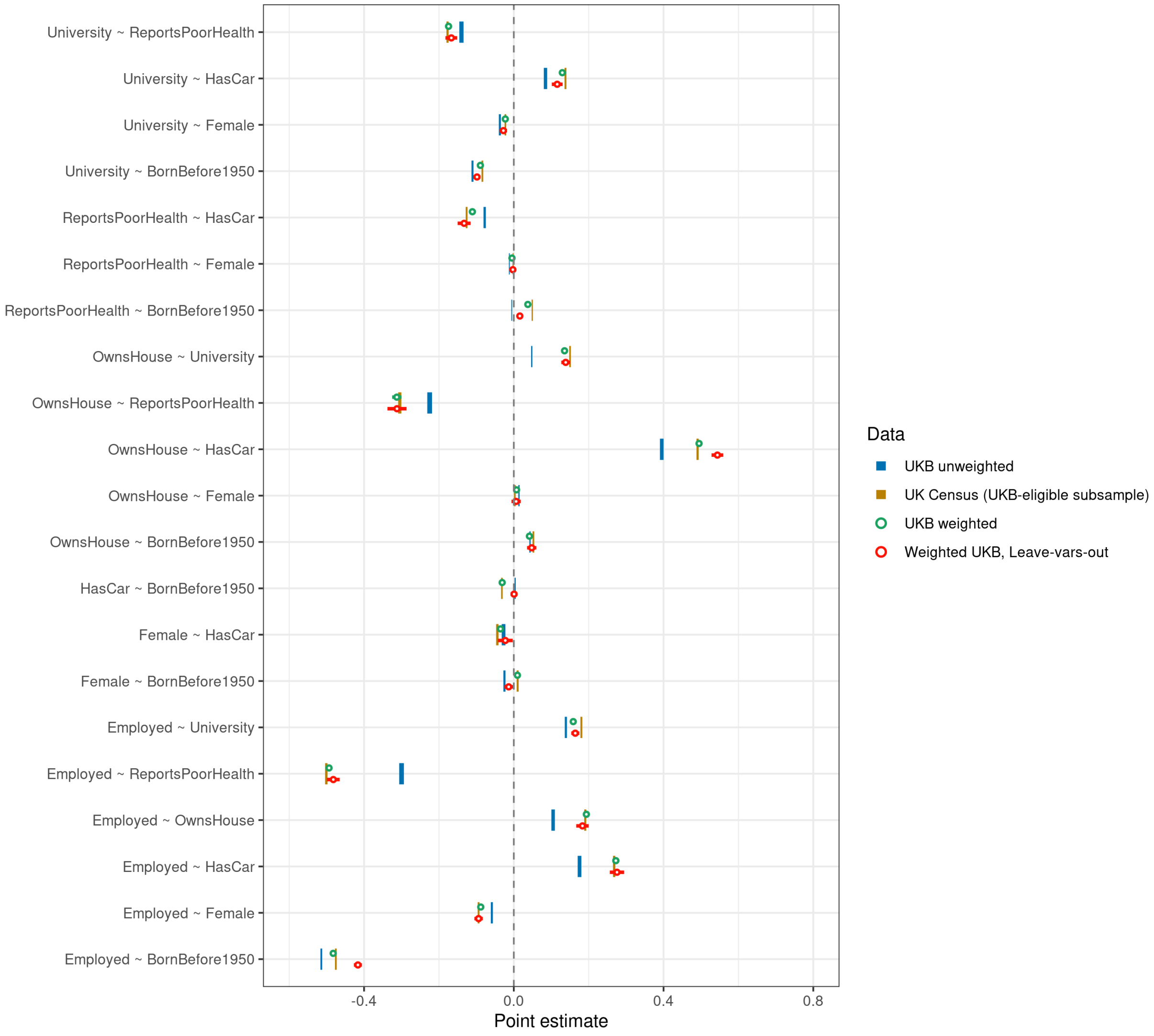

Figure S4: Estimated coefficients for bivariate linear models in UKB and UK Census when leaving relevant model variables out of IP-weight construction. Associations are as shown in figure 3. Additionally, the red open circles show the results from weighted models in the UKB that are based on "leave-variables-out" weights. These weights are constructed in the same manner as the IP weights, but are based on a LASSO model that did *not* include the dependent and independent variable included in the association model shown. All results estimated on the first holdout sample only.

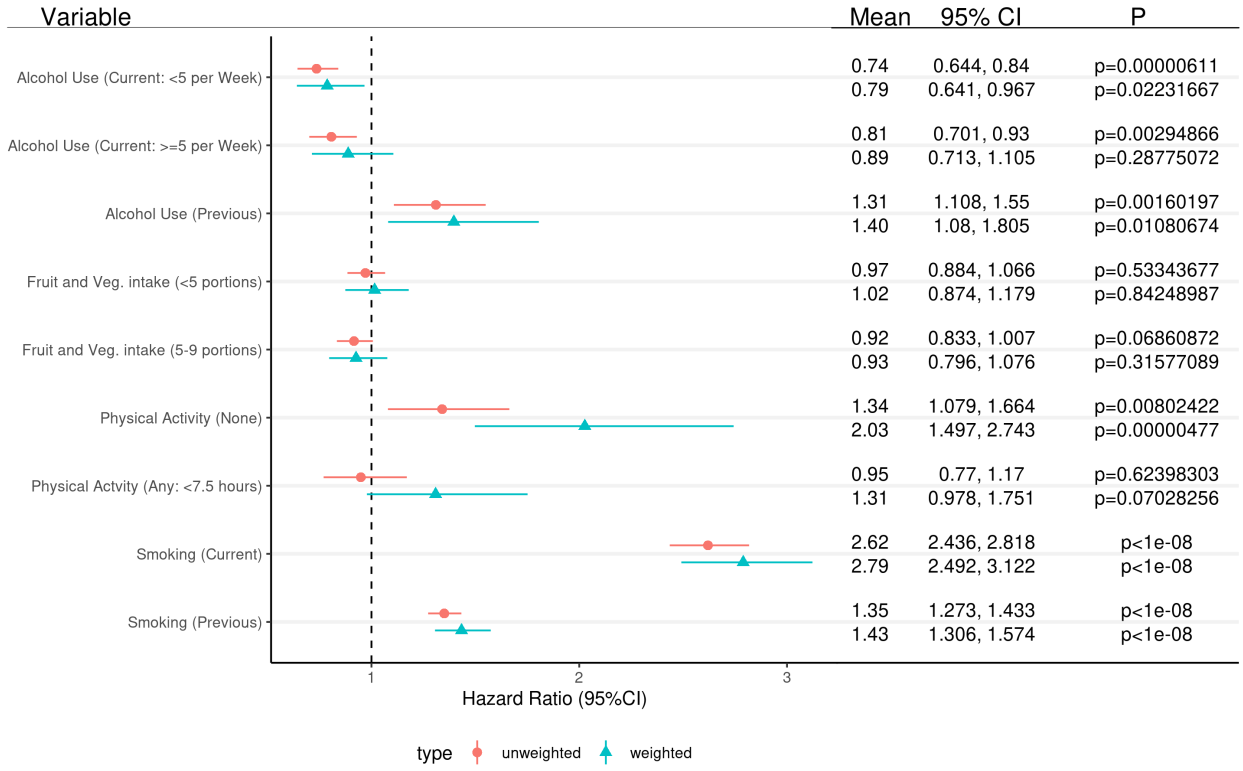

Figure S5: Cox hazard ratio estimates of all-cause mortality mutually adjusting for various lifestyle factors. The hazard ratios indicated by the round dot (in red) are unweighted, whereas the hazard ratios indicated by the triangle (in blue) are obtained from a weighted model that uses weights inverse proportional to UKB participation, estimated using UK Census data. The model is mutually adjusted for sex and highest educational qualification, and controls for initial age at baseline. The excluded reference categories are: “No alcohol use‘’, “more than 10 portions of fruit and vegetable intake per day”, “more than 7.5 hours of physical activity per week”, “and never smoked”. Those with missing values on any of the included variables, and those with a prior history of cardiovascular disease or cancer before baseline were excluded (N = 323,598 after exclusion).

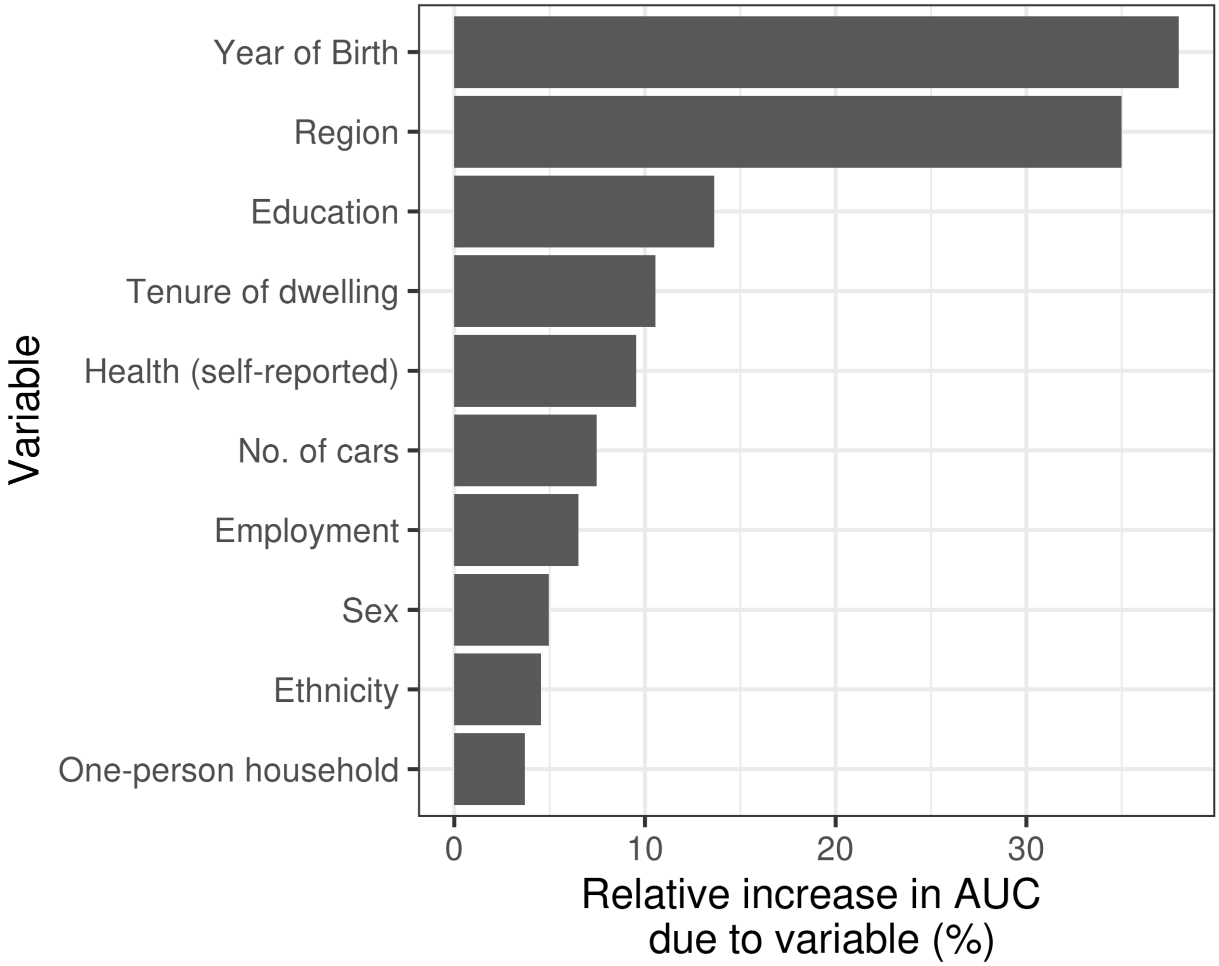

Figure S6: Variable importance plot showing the importance of each variable in predicting UKB volunteering on the first holdout sample. Variable importance is assessed by the relative increase in the AUC, relative to the full model's AUC. The variable importance plots for the LASSO model estimated on data that holds out the 4 other folds look very similar and are therefore not shown here.

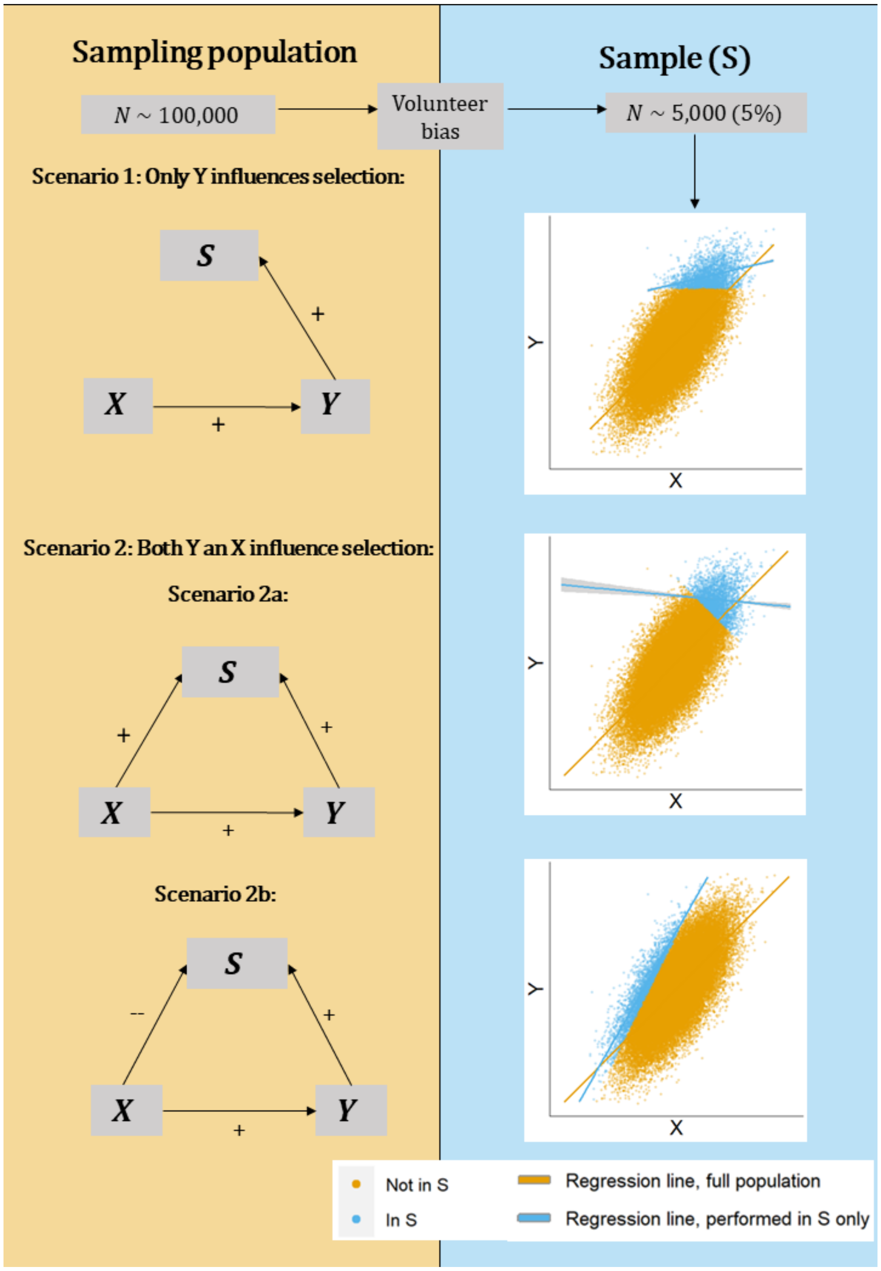

Figure S7: A simulated example of spurious associations due to volunteer bias in a selected sample S. In this example, we simulate an exposure $X \sim \mathcal{N}(0,1)$ and an outcome $Y = X + \epsilon$, $\epsilon \sim \mathcal{N}(0,1)$. X and Y are positively related in the population (the orange and blue dots combined) with slope 1. This is reflected by the orange regression lines in each of the three scatter plots. In scenario 1, individuals with higher values of Y, here modelled by a threshold $Y > Y^*$, select into the sample (S; the blue points) and there is no selection based on X. As a result, the regression line estimated within the selected sample S (the blue line) is attenuated towards the null. In scenario 2a, individuals with higher values of Y and with higher values of X, here modelled by a threshold $0.5Y + 0.5X > Z^*$, select into the sample S. As a result, the regression is downwards biased (and of incorrect sign). In scenario 2b, individuals with higher values of Y, but lower values of X, select into the sample S (here modelled by a threshold $Y - 2X > Z^*$). Now, the bias is upwards, and the effect of X on Y is overestimated.

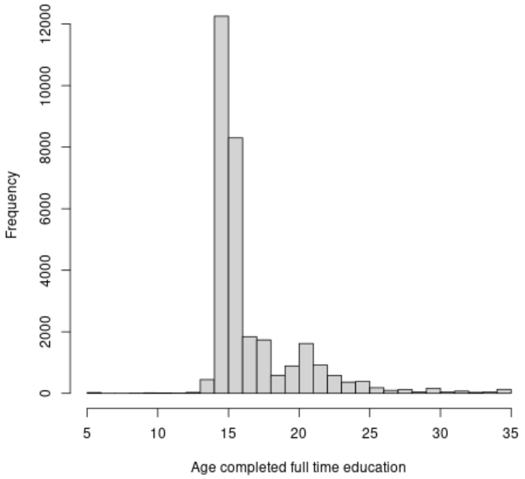

(a) Distribution of “age completed full time education”, for those with an NVQ or HNC or HND or equivalent

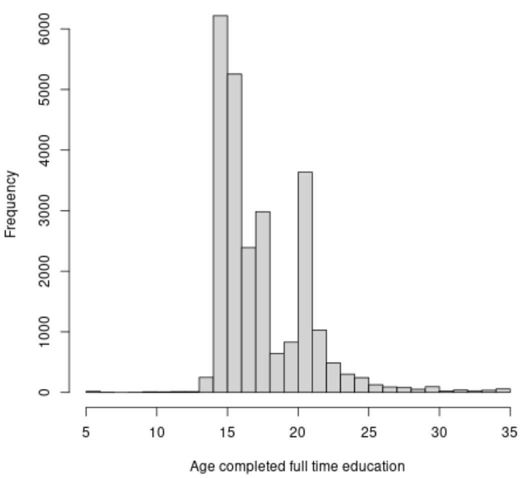

(b) Distribution of “age completed full time education”, for those with a professional qualification not elsewhere classified

Figure S8

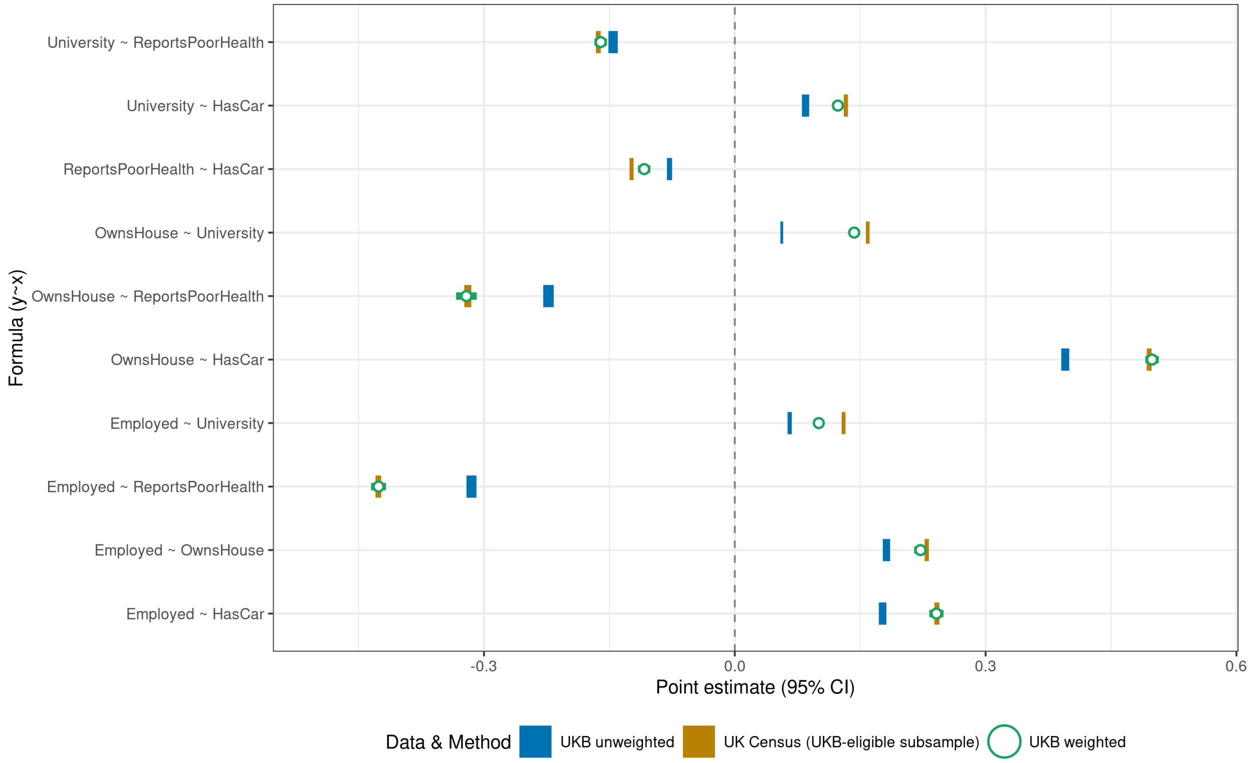

Figure S9: Estimated associations based on bivariate linear models in UKB (solid blue bars), UKB-eligible Census (solid yellow bars), and Weighted UKB (open green circles) (as in Figure 3), after (linearly) controlling for year of birth and sex. Bar widths indicate 95% confidence intervals (heteroskedasticity-robust standard errors). All blue and yellow bars are highly significantly different from one another ($P < 10^{-8}$).

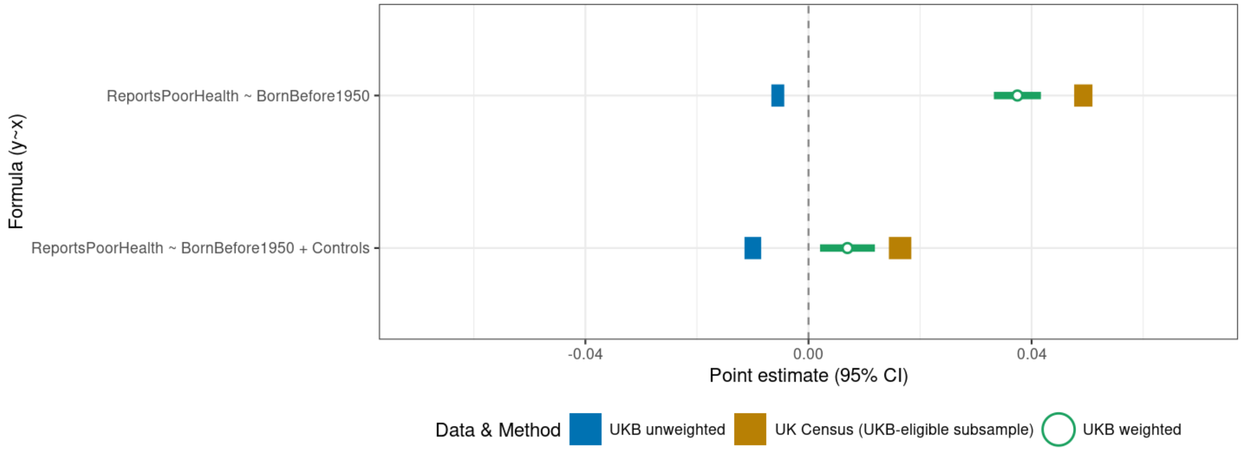

Figure S10: Estimated coefficients for the effect of being born before 1950 on reporting poor health in UKB (solid blue bars), UKB-eligible Census (solid yellow bars), and Weighted UKB (open green circles). The first model shown (ReportsPoorHealth ~ BornBefore1950) does not include any control variables and is the same as the one reported in figure 3. The second model (ReportsPoorHealth ~ BornBefore1950) + Controls) shows the same coefficients after adding various control variables to the regression: sex, years of education, number of cars owned, a single household indicator, tenure of household (4 dummy variables), employment status (5 dummy variables), ethnicity (4 dummy variables), and region (142 dummy variables).

References

1 Breen R, Ermisch J. Using Inverse Probability Weighting to Address Post-Outcome Collider Bias. Sociological Methods & Research. 2021:00491241211043131.

2 Lu H, Cole SR, Howe CJ, Westreich D. Toward a clearer definition of selection bias when estimating causal effects. Epidemiology. 2022;33(5):699-706.

3 Fry A, Littlejohns TJ, Sudlow C, Doherty N, Adamska L, Sprosen T, et al. Comparison of sociodemographic and health-related characteristics of UK Biobank participants with those of the general population. American journal of epidemiology. 2017;186(9):1026-34.

4 Lee JJ, Wedow R, Okbay A, et al. Gene discovery and polygenic prediction from a genome-wide association study of educational attainment in 1.1 million individuals. Nature genetics. 2018;50:1112-21.

5 Education GPS;. Accessed: 2022-02-11. https://gpseducation.oecd.org/CountryProfile?primaryCountry=GBR&treshold=10&topic=EO.

6 Okbay A, Wu Y, Wang N, et al. Polygenic prediction of educational attainment within and between families from genome-wide association analyses in 3 million individuals. Nature genetics. 2022;54:437-49.

7 Tibshirani R. Regression shrinkage and selection via the lasso. Journal of the Royal Statistical Society: Series B (Methodological). 1996;58:267-88.

8 Hastie T, Qian J. Glmnet vignette; 2014. http://www.web.stanford.edu/~hastie/Papers/Glmnet_Vignette.pdf.

9 Robin X, Turck N, Hainard A, et al. pROC: an open-source package for R and S+ to analyze and compare ROC curves. BMC bioinformatics. 2011;12:1-8.

10 Domingue B, Rahal C, Faul J, Freese J, Kanopka K, Rigos A, et al. InterModel Vigorish (IMV): A novel approach for quantifying predictive accuracy with binary outcomes. SocArXiv. 2021.

11 Domingue BW, Belsky DW, Harrati A, Conley D, Weir DR, Boardman JD. Mortality selection in a genetic sample and implications for association studies. International Journal of Epidemiology. 2017;46:1285-94.

12 Robins JM, Hernan MA, Brumback B. Marginal structural models and causal inference in epidemiology. Epidemiology. 2000.

13 Hernán MA, Hernández-Díaz S, Robins JM. A structural approach to selection bias. Epidemiology. 2004:615-25.

14 Cole SR, Hernán MA. Constructing inverse probability weights for marginal structural models. American journal of epidemiology. 2008;168:656-64.

15 Stamatakis E, Owen KB, Shepherd L, Drayton B, Hamer M, Bauman AE. Is cohort representativeness Passé? Poststratified associations of lifestyle risk factors with mortality in the UK Biobank. Epidemiology. 2021;32:179.

16 Potthoff RF, Woodbury MA, Manton KG. "Equivalent sample size" and "equivalent degrees of freedom" refinements for inference using survey weights under superpopulation models. Journal of the American Statistical Association. 1992;87:383-96.

17 Howe LJ, Nivard MG, Morris TT, Hansen AF, Rasheed H, Cho Y, et al. Within-sibship GWAS improve estimates of direct genetic effects. bioRxiv. 2021.

18 Pirastu N, Cordioli M, Nandakumar P, Mignogna G, Abdellaoui A, Hollis B, et al. Genetic analyses identify widespread sex-differential participation bias. Nature Genetics. 2021;53(5):663-71.
